# Influence of Harvesting and Seasonal Variability on the Physicochemical and Antioxidant Properties of Native Bee *(Tetragonisca fiebrigi)* Honey from Bolivia’s Tropical Dry Forests

**DOI:** 10.3390/molecules31111819

**Published:** 2026-05-25

**Authors:** Alejandra Romero-Padilla, Luís M. G. Castro, Manuela Pintado, María Emilia Brassesco

**Affiliations:** 1 Biotechnology, Universidad Católica Boliviana San Pablo, Santa Cruz, Bolivia; alejandra.romero2201@gmail.com; 2 Universidade Católica Portuguesa, CBQF—Centro de Biotecnologia e Química Fina—Laboratório Associado, Escola Superior de Biotecnologia, Rua Diogo Botelho 1327, 4169-005 Porto, Portugal; lgcastro@ucp.pt (L.M.G.C.); mbrassesco@ucp.pt (M.E.B.)

**Keywords:** stingless bee honey, harvesting methods, antioxidant activity, seasonal variability, Bolivia, physicochemical characterization

## Abstract

This study evaluates the influence of harvesting methods and seasonal variability on the physicochemical and antioxidant properties of *Tetragonisca fiebrigi* honey produced in the tropical dry forest of Bolivia. Despite the growing interest in stingless bee honey, studies addressing the combined effects of seasonality and collection practices in this region remain scarce. Honey samples were collected during winter and spring using three approaches: conventional, optimized (based on good manufacturing practices), and direct racking from natural nests. Physicochemical parameters (pH 4.60–6.15; moisture 28–34%; water activity 0.69–0.75) and sugar composition (glucose 10.60–29.03 g/100 g; fructose 9.01–21.97 g/100 g; sucrose 0.70–3.23 g/100 g) showed variability primarily associated with season rather than harvesting method. Bioactive compounds exhibited a marked seasonal effect, with higher total phenolic content (up to 11.03 mg GAE/100 g), flavonoids (up to 23.08 mg QE/100 g), and antioxidant capacity (DPPH up to 1.33 mol TE/100 g; ORAC up to 25.93 mol TE/100 g) in spring samples. Multivariate analysis (PCA) revealed that honey variability is structured along bioactive and physicochemical axes, with samples obtained using the optimized method showing reduced dispersion and greater compositional consistency. These results indicate that while seasonality governs the compositional and functional properties of *T. fiebrigi* honey, improved harvesting practices contribute to reducing variability and enhancing product standardization. This study provides one of the first comprehensive datasets on Bolivian stingless bee honey and highlights its potential as a functional food, supporting the development of species-specific quality criteria and sustainable meliponiculture in tropical dry forest ecosystems.

## 1. Introduction

Stingless bee honey (SBH) is increasingly recognized for its nutritional, medicinal, and sensory value. Unlike honey from the Western honey bee (*Apis mellifera*), which dominates global apiculture, SBH is produced by bees of the *Meliponini* tribe and exhibits distinct characteristics—such as higher moisture and acidity, greater electrical conductivity, and a sour–sweet flavor. These differences stem not only from species-specific physiology, but also from the unique method of honey storage in wax-resin pots, where physical, chemical, and microbial processes modify the honey composition during maturation [1,2].

Beyond its sensory appeal, SBH possesses notable bioactive properties. It demonstrates up to 45% greater antioxidant and antimicrobial activity compared to *A. mellifera* honey, along with reported anti-inflammatory, antidiabetic, and wound-healing effects [3,4]. These attributes are largely influenced by factors such as geographic origin, floral sources, and environmental conditions, which vary across regions and species [5].

In Bolivia, SBH has long been valued in traditional medicine, particularly in regions like the Chaco and the tropical dry forests. However, scientific research on the composition and quality of Bolivian SBH remains lacking [6]. Among the native stingless bees, *Tetragonisca fiebrigi*—locally known as “Señorita”—is one of the most culturally and economically important species. Its honey is traditionally used to treat respiratory and digestive complaints and is prized for its fluid texture and acidic-sweet flavor. Despite its popularity, its physicochemical and biological properties remain poorly characterized [4,7,8].

Comparative studies conducted in Argentina and Paraguay have shown that *T. fiebrigi* honey contains flavonoid concentrations ranging from 4.40 to 45.44 mg QE/100 g, far exceeding those found in *A. mellifera* honey (≈2.5 mg/100 g) (QE = Quercetin Equivalent). While polyphenol content across different regions remains relatively stable, the antioxidant capacity of *T. fiebrigi* honey has been reported to reach values as high as 323.70 mmol TE/100 g (TE = Trolox Equivalent), with no direct correlation to total phenolic content—suggesting the presence of alternative bioactive mechanisms. In addition, its antimicrobial activity appears to be selective, showing stronger inhibition of *Staphylococcus aureus* compared to *Escherichia coli*, and even supporting the growth of beneficial microbial species under certain conditions. Previous studies have reported associations between stingless bee honey and metabolically active *Bacillus* species, including *Bacillus alvei*, *Bacillus circulans*, and *Bacillus megaterium*, which are known to produce enzymes, antifungal compounds, fatty acids, and antibiotic-like substances that may contribute to food preservation, increased digestibility, and the antimicrobial properties of the honey [9].

Although recent Bolivian studies have documented the pollen spectrum and seasonal variability of SBH, a key gap remains: the impact of harvesting methods and seasonality on honey quality. This is especially critical in artisanal settings, where non-standardized or unhygienic practices can compromise honey composition, accelerate fermentation, and reduce both shelf life and bioactive potency [10].

In this context, parameters such as pH, moisture, sugar content, and antioxidant capacity are essential for assessing honey freshness, stability, and therapeutic value. However, these variables may fluctuate widely depending on bee species, environmental conditions, and harvest techniques [11,12]. For example, SBH can present moisture levels as high as 32%, contributing to microbial instability if not properly handled. The acidity, primarily from organic acids and natural fermentation, contributes both to antimicrobial action and self-preservation [13].

Despite its nutritional and therapeutic promise, *T. fiebrigi* honey faces major barriers to standardization and commercialization due to the lack of scientific data and harvesting protocols tailored to Bolivia’s tropical dry forest ecosystem.

This study addresses this critical gap by evaluating the effect of different honey harvesting methods and seasonal variation on the physicochemical and biological properties of *Tetragonisca fiebrigi* honey. It represents one of the first scientific investigations on this species in Bolivia and aims to provide foundational data to support its valorization as a functional food, the development of quality standards, and the promotion of sustainable, conservation-oriented meliponiculture in the region.

## 2. Results

### 2.1. Physicochemical Characteristics

The physicochemical properties of *Tetragonisca fiebrigi* honey were assessed in relation to both harvesting methods and season. The results are presented in Table 1.

pH values differed significantly among samples (*p* ≤ 0.05), ranging from 4.59 to 6.15, confirming the naturally acidic character of *Tetragonisca fiebrigi* honey [14]. In general, all winter samples—regardless of harvesting method—exhibited lower pH values, although significant variation was observed between individual nests without following a consistent pattern. This suggests that hive-specific factors may play a role in modulating acidity, such as floral availability, nectar composition, or environmental conditions specific to the dry season [15,16]. In contrast, spring samples showed overall higher pH values, with notable variation depending on the harvesting method: the lowest pH in spring was observed in the conventional sample (PC1: 5.07 ± 0.04), while the highest was found in the racking method (PT2: 6.15 ± 0.01). These results suggest that both seasonal changes and the type of harvesting method may modulate the acidity of the honey, potentially through effects on nectar composition, microbial exposure, or fermentation dynamics during collection and storage [17]. Variations in pH may also be associated with enzymatic activity during honey maturation, particularly glucose oxidase activity, as well as microbial fermentation processes in high-moisture environments characteristic of stingless bee honey [18]. Inadequate handling or insufficient sterilization during collection may introduce environmental microorganisms such as yeasts and bacteria, promoting the production of organic acids and consequently modifying pH values [18,19]. In contrast, optimized harvesting methods that selectively collect mature honey and minimize contamination may contribute to more stable physicochemical characteristics.

Ash content, which reflects the mineral composition of honey, was generally consistent across most samples (Table 1), with no significant differences observed among them. Values ranged from 11.96 to 12.65 g/100 g, regardless of season or harvesting method, suggesting a stable mineral profile largely unaffected by external factors. This overall stability is consistent with previous findings indicating that the mineral composition of honey is primarily influenced by soil characteristics and botanical origin, as minerals are absorbed by plant roots, transferred to the nectar, and ultimately incorporated into honey [20].

However, one notable exception was observed in a spring sample collected by direct racking (PT2: 6.15 ± 0.28 g/100 g), which showed a markedly lower ash content. This deviation may be associated with colony-specific conditions, differences in floral sources, or environmental factors affecting mineral uptake. It is well established that potassium is the most abundant mineral in honey, followed by calcium and magnesium, while trace elements such as zinc, phosphorus, manganese, lead, iron, and sodium may also be present [20,21,22]. These variations in mineral composition further support the influence of botanical and environmental factors on the ash content of honey.

Moisture content was consistently within the range of 28–34% across most samples, aligning with typical values reported for stingless bee honey, which generally exhibits higher moisture than *Apis mellifera* honey and can reach up to 40% [3,21]. These results are also consistent with the average moisture content of approximately 31% reported in previous studies [16].

The relatively uniform values observed suggest that harvesting and storage practices were effective in preserving honey stability and quality-related components, including organic acids, enzymes, and minerals. Since moisture content is a key factor influencing microbial activity and shelf life [13], the results support the overall physicochemical quality of the analyzed samples [22].

Protein content varied substantially among samples, ranging from 1.32 to 16.35 g/100 g, indicating a strong influence from both seasonality and harvesting methods. The lowest values were recorded in winter samples collected using the optimized method (IO1–IO2: 1.32–1.58 g/100 g), while the highest protein concentrations were observed in spring samples obtained via racking and optimized collection (PT1: 16.35 g/100 g; PT2: 16.2 g/100 g). Intermediate values were found in both winter and spring samples collected by conventional means or other combinations, suggesting a multifactorial influence on protein levels. Notably, some winter samples collected through the optimized method shared similar floral origins and exhibited comparable protein concentrations, suggesting that botanical source and nectar composition—alongside bee-secreted enzymes—may play a central role in determining protein content [23,24,25]. These findings underscore the relevance of season, floral diversity, and handling practices in shaping the nutritional quality of stingless bee honey. In this study, water activity (Aw) values ranged from 0.69 to 0.75 (Table 1), which are slightly above the ranges typically reported for honey (~0.50–0.65) yet remain below critical thresholds for bacterial proliferation (>0.75) [26]. These values suggest a relatively low susceptibility to microbial contamination, but their proximity to the upper limit underscores the importance of other protective factors—particularly the naturally low pH of the samples (4.59–6.15), which enhances microbial stability. Seasonal and methodological influences were evident: the highest Aw values occurred in winter samples collected using the optimized method, while the lowest values were observed in spring samples obtained by direct racking. Importantly, as shown by Zamora et al. (2006) [27], there is a non-linear relationship between moisture content and Aw, mediated by sugar composition. During crystallization, glucose tends to solidify first, releasing water that increases Aw. This process has implications for honey stability, shelf life, and vulnerability to microbial spoilage [1]. Our findings reflect this: samples with higher moisture percentages (e.g., IO1, IO2) also tended to exhibit elevated Aw, supporting the idea that both nectar composition and environmental humidity contribute to free water availability. This pattern indicates that both seasonal factors and extraction methods may influence the water content in honey, in line with previous studies that emphasize the impact of botanical origin, climate, and collection technique on moisture levels [25].

Color measurements revealed notable variations across samples, reflecting the influence of seasonality, extraction method, and potential storage conditions. Lightness (L*) values ranged from 58.06 to 69.02, with winter samples generally exhibiting higher L* (e.g., IC3: 69.02 ± 0.07) and spring samples tending toward lower L* (e.g., PO1: 58.18 ± 0.17), suggesting that darker honeys are associated with spring collection or certain extraction practices. Differences in chromatic coordinates (a* and b*) further illustrated these trends: a* values, representing the red–green axis, were highest in some winter and spring samples (e.g., IC1: 2.93 ± 0.04; PT2: 2.20 ± 0.05), while b* values, representing the yellow–blue axis, ranged from 4.70 to 12.99, with the darkest honeys (lower L*) displaying higher b* values, consistent with elevated mineral and sugar content.

These observations are consistent with prior studies indicating that darker honeys generally contain higher phenolic content [28], and samples from Alta Vista confirmed this correlation: lower L* (darker color) corresponded to increased total phenolic compounds, suggesting that color intensity can serve as an indirect indicator of bioactive compound levels [29]. Overall, the color analysis highlights that both season and extraction methods influence honey appearance, which has practical implications for consumer preference and product marketability in the beekeeping industry.

Finally, total phenolic compounds (TPC) and total flavonoids were quantified to assess the antioxidant potential of the honey samples. Both parameters showed significant variability across samples, influenced primarily by seasonal floral diversity and, to a lesser extent, by harvesting methods. TPC values ranged from 3.17 to 11.03 mg GAE/100 g, with the highest concentrations found in spring racking samples (PT1, PT2)—likely due to greater floral resource availability and botanical diversity during this season. Conversely, winter samples collected using either the optimized (IO3, IO4) or conventional method (IC3) exhibited the lowest TPC values, reflecting more limited floral input and possibly reduced synthesis of phenolic precursors [30]. Flavonoid content followed a comparable seasonal trend, ranging from 10.21 to 23.08 mg QE/100 g, with higher values again associated with spring racking samples, reinforcing the idea that floral origin and seasonal phenology are critical determinants of secondary metabolite profiles. These findings align with previous studies indicating that stingless bee honeys exhibit considerable variation in antioxidant compounds due to differences in nectar composition and local vegetation. Phenolic compounds in SBH, including flavonoids, phenolic acids, and tannins, are primarily derived from nectar and pollen collected from diverse plant sources, which explains the observed variability [15,31].

The higher TPC and flavonoid values observed in spring samples may therefore be associated with increased floral diversity and resource availability during this season. These values fall within the variability range reported for stingless bee honey in the literature.

### 2.2. Sugar Composition

Sugars are the major constituents of honey, typically accounting for 70–80% of its weight, and are critical determinants of its nutritional value, sweetness, fermentability, and crystallization behavior. The principal monosaccharides—glucose and fructose—are derived from the enzymatic hydrolysis of sucrose by invertase secreted by bees. Their relative proportions vary according to botanical origin, bee species, climatic conditions, and maturity at harvest, making sugar profiling a key parameter in honey characterization [18,32,33].

In the present study, sugar composition varied significantly across the eleven analyzed samples, reflecting a combined influence of season, extraction method, and inter-colony variability (Table 1). Glucose content ranged from 10.60 to 29.03 g/100 g, with the highest levels found in spring samples—notably PO1 (29.03 ± 2.12) and PC1 (25.72 ± 1.41)—likely due to increased nectar availability during flowering peaks. In contrast, lower glucose levels were recorded in winter samples, such as IO2 (10.60 ± 0.98) and IC1 (12.65 ± 1.10).

Fructose concentrations ranged from 9.01 to 21.97 g/100 g, with IC2 (21.97 ± 1.14) and PO1 (21.02 ± 1.82) exhibiting the highest values. Conversely, IO2 (9.01 ± 0.67) showed the lowest fructose concentration, consistent with reduced floral availability and altered nectar composition in the dry season. Compared to literature values for Apis mellifera honeys, which report average fructose contents around 31.7 g/100 g, these values are notably lower—likely reflecting species-specific differences in sugar metabolism and foraging behavior [34].

The glucose-to-fructose ratio, a critical factor in crystallization tendency, also varied across samples. In general, winter samples—particularly those obtained using the optimized method (IO1, IO2)—showed a more balanced ratio, potentially reducing crystallization rates and helping maintain honey fluidity and stability [35].

Sucrose content was comparatively low across all samples, ranging from 0.70 to 3.23 g/100 g. However, higher values were noted in spring racking samples such as PO1 (3.23 ± 0.29) and PT1 (2.36 ± 0.18). Elevated sucrose levels in these samples may indicate incomplete enzymatic hydrolysis or early-stage harvests, as well as minimal handling and filtration during racking, which could retain more unconverted sugars. Despite this, all sucrose values remain within acceptable limits for stingless bee honeys (typically <5%), supporting the natural maturity and authenticity of the samples [34].

Interestingly, even within the same season and collection method, considerable variation was observed among individual colonies. For instance, glucose levels in spring samples ranged from 15.83 (PT2) to 29.03 g/100 g (PO1), highlighting colony-specific factors such as foraging range, floral preference, and hive microenvironment as important contributors to sugar composition. This underscores the complexity of native honey production systems and the need for colony-level quality monitoring in meliponiculture practices.

Overall, these results demonstrate that seasonal conditions, floral dynamics, and even intra-species variability significantly influence sugar composition in *Tetragonisca fiebrigi* honey. Understanding these factors is essential for evaluating honey stability, shelf life, fermentation risk, and consumer acceptance, particularly in the development of regionally standardized products.

### 2.3. Antioxidant Properties

Biological activities were assessed to evaluate the antioxidant potential of native stingless bee honey using phenolic and flavonoid content as well as multiple assays (DPPH, ORAC, ABTS+). The results of each methodology for each honey sample is presented in Table 2. The use of complementary methods allowed for cross-verification of results, reducing assay-specific bias and providing a more robust characterization of the honey’s bioactive profile. Emphasis was placed on phenolic compounds and flavonoids, given their key role in oxidative stability and bioactivity [3].

ABTS+ and ORAC assays indicated the highest activity in spring racking or optimized samples, while winter samples generally showed lower activity. DPPH results were more uniform but still reflected slightly higher activity in spring-collected honeys (Table 2).

A positive correlation was evident between phenolic/flavonoid content and antioxidant capacity, as exemplified by PT2 (the highest phenolics and strong antioxidant activity) versus IO3 (the lowest phenolics and reduced activity). Furthermore, differences among extraction methods suggest that racking or optimized techniques help preserve bioactive compounds, likely by minimizing mechanical stress or exposure to oxygen [36,37]. Seasonal differences also indicate that spring floral diversity not only increases phenolic and flavonoid content but may enhance synergistic effects, resulting in higher antioxidant performance across multiple assays.

These patterns suggest that both the chemical composition and the bioactivity of honey are shaped by harvest timing and method, which may have practical implications for honey quality, shelf-life, and health-promoting properties [38,39]. For example, honeys with higher phenolic content and antioxidant capacity may be more resistant to oxidation and enzymatic degradation during storage, supporting their potential nutritional and commercial value.

Overall, both harvest season and extraction method significantly influence antioxidant capacity, with spring-collected racking and optimized samples consistently exhibiting enhanced bioactive profiles. These findings underscore the importance of floral source, seasonality, and extraction practice in determining the quality, stability, and potential health benefits of native stingless bee honey [40].

### 2.4. Principal Component Analysis (PCA)

To understand the collective behavior of variables, a PCA was conducted.

**Figure 1:**
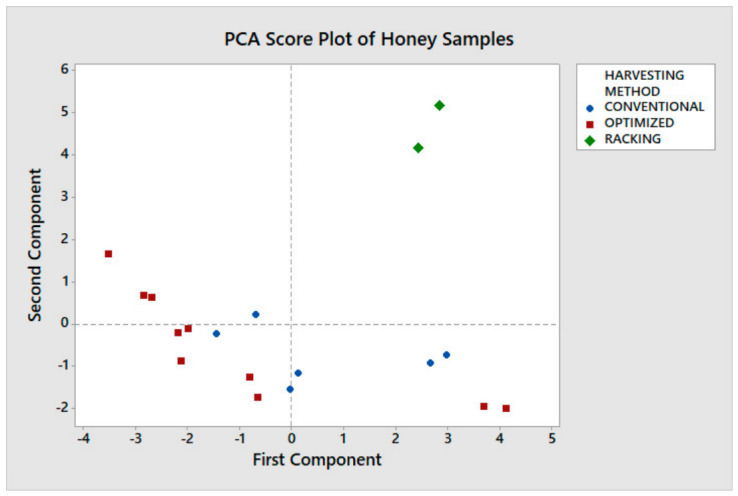
PCA score plot showing the distribution of honey samples according to harvesting method and season based on physicochemical and antioxidant parameters.

In Figure 1, the score plot provides a visualization of sample groupings based on the multivariate distribution of the analyzed parameters. It shows that samples obtained using the optimized method display more pronounced clustering, whereas those obtained through the conventional method show greater dispersion, suggesting higher variability in physicochemical properties. Meanwhile, samples obtained by direct racking method appear scattered in a separate region from the other harvesting methods, indicating a distinct compositional profile.

**Figure 2:**
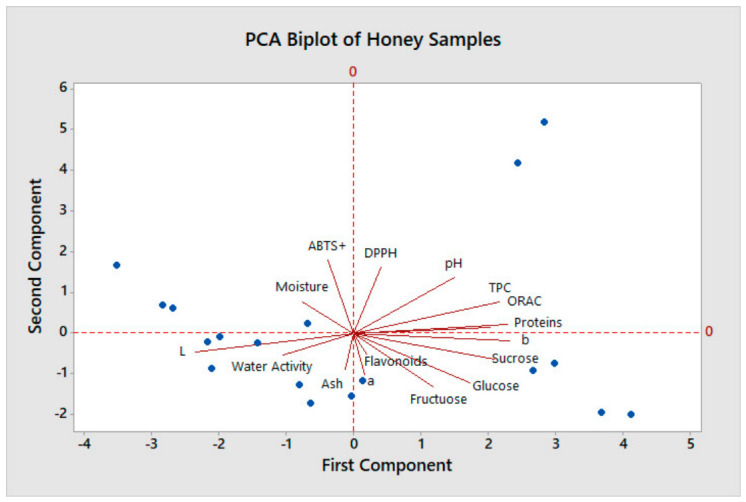
PCA biplot illustrating the contribution of physicochemical and biological variables to sample distribution along PC1 and PC2. Blue dots represent individual honey samples. The direction and length of the line indicate the contribution of each variable to the principal components.

The biplot, shown in Figure 2, illustrates the influence of different variables in the analysis. Variables with the highest contribution to PC1 included phenols, proteins, and ORAC values, whereas variables such as ABTS^+^ and DPPH contributed more significantly to PC2. PC1 explained 38.5% of the total variability, while the first two principal components together accounted for 62.9% of the total variance, indicating that the PCA captured a substantial proportion of the variability within the dataset. In addition, variables such as pH, sugars, phenols, and antioxidant activity showed important contributions to sample differentiation. Detailed PCA outputs, including explained variance and variable contributions, are provided in the Supplementary Material.

Despite these insights, it is important to acknowledge certain limitations. Variations in environmental conditions such as temperature, humidity, and floral source during collection were not fully standardized and may have influenced the results. Additionally, the relatively small sample size limits the generalizability of the observed clustering patterns.

Nonetheless, the results suggest that optimized collecting methods may contribute to improved compositional consistency and reduced sample variability, which could be relevant for future quality assurance strategies. These findings may also support the development of regional collection guidelines and certification systems, contributing to the valorization and sustainable commercialization of native stingless bee honey in Bolivia.

## 3. Discussion

The present study provides one of the first integrated physicochemical and functional characterizations of *Tetragonisca fiebrigi* honey from Bolivia’s tropical dry forest. Overall, the results indicate that both seasonality and harvesting practices influence honey quality, although seasonality appears to exert the strongest effect on compositional and bioactive parameters, whereas optimized harvesting mainly contributes to reduced variability and improved sample homogeneity.

These findings complement recent research on Bolivian honeys. J.A. Limpias-Hurtado et al. reported significant variability in the phenolic composition and antioxidant capacity of *Apis mellifera* honeys from different ecoregions of Santa Cruz, demonstrating that local flora, harvest season, and beekeeping practices are key determinants of honey bioactivity. While Limpias focused on *A. mellifera*, the present study extends this regional understanding to stingless bee honey, highlighting the broader ecological and geographical drivers shaping the functional properties of Bolivian honeys [41].

The first relevant aspect is the acidity profile. The pH values observed here (4.59–6.15) confirm the naturally acidic character of stingless bee honey, although they are somewhat higher than those commonly reported for many *Apis mellifera* honeys and for some stingless bee honeys from other tropical regions. Nordin et al. described stingless bee honey as typically more acidic than *A. mellifera* honey, with pH usually between 3.2 and 4.5, although substantial interspecific and geographical variability exists [24]. Similar ranges were reported by Sant’Ana et al. for Brazilian stingless bee honeys [42], while Schvezov et al. also showed lower pH values for *Tetragonisca fiebrigi* honey from Argentina [7]. Therefore, the comparatively higher pH values recorded in some spring samples in the present study likely reflect regional floral composition, climatic conditions, and species-specific maturation dynamics rather than reduced product quality. This interpretation is supported by the fact that winter samples, despite significant nest-to-nest variation, remained grouped within the lower pH range, suggesting that seasonal nectar composition and environmental constraints during the dry period may favor a more acidic profile.

The relationship between acidity and microbiological stability should also be interpreted together with water availability. Stingless bee honey is well known to have higher moisture and water activity than conventional honey [17,24]. In the present work, Aw values ranged from 0.69 to 0.75, which are above those typically found in *A. mellifera* honeys but remain consistent with reports for stingless bee honey from Southeast Asia and Latin America [5,16,22] Ismail et al. and Chuttong et al. emphasized that free water availability in stingless bee honey depends strongly on botanical origin, dehumidification capacity inside the nest, and post-harvest handling [16,22]. Similarly, Zamora et al. demonstrated that the relationship between moisture and water activity in honey is non-linear and highly dependent on sugar composition, especially glucose crystallization behavior [27]. Thus, the Aw values reported here should not be interpreted as evidence of instability per se, but rather as part of the expected physicochemical profile of pot honey. In this context, the coexistence of moderate Aw, acidic pH, and high sugar concentration likely acts as a combined hurdle system limiting microbial growth. This interpretation is also in line with the broader view proposed by Ávila et al., who noted that quality assessment in stingless bee honey must consider multiple interacting parameters rather than applying criteria derived from *A. mellifera* honey alone [17].

Moisture content further supports this distinction. The values observed in this study are within the range frequently reported for stingless bee honey and are notably higher than the legal thresholds established for *A. mellifera* honey. Bijlsma et al. showed that honey from stingless bees may naturally present markedly higher water content due to species-specific storage biology and climatic conditions [12], while Nordin et al. reported wide global variation in moisture among stingless bee honeys [24]. Malaysian and Brazilian studies have likewise shown that moisture values around 25–35% are not uncommon in these honeys [5,13,22,43]. Therefore, the moisture content observed here should be interpreted as a species-related trait rather than a sign of poor handling. This is particularly relevant from a regulatory standpoint, as it reinforces the need for species-specific quality standards for Bolivian stingless bee honey.

Sugar composition provides additional evidence of the distinct physicochemical identity of *T. fiebrigi* honey. Glucose and fructose were the major sugars, as expected, but several samples showed relatively balanced concentrations or even a slight predominance of glucose. This differs from the classic profile of *A. mellifera* honey, in which fructose usually predominates [34]. Similar deviations have already been reported for stingless bee honeys, whose sugar profiles depend strongly on floral source, bee species, and post-secretory transformations inside the nest [25,29]. De Sousa et al. showed that monofloral stingless bee honeys from Brazil may vary substantially in glucose/fructose ratios depending on species and environmental context [25], while Julika et al. reported broad variation in sugar composition for Malaysian stingless bee honey [11]. In the present study, spring samples tended to exhibit higher glucose and fructose levels, supporting the interpretation that increased nectar availability and floral diversity during the blooming season contribute to greater sugar accumulation. At the same time, marked inter-colony variation was evident even within the same season and harvesting method, highlighting the importance of colony-specific factors such as foraging behavior, floral preference, and microenvironment. This is consistent with the notion that variability is a defining characteristic of stingless bee honey systems [17,24].

Sucrose levels remained low in all samples, which supports the natural maturity and authenticity of the honeys analyzed. According to international criteria discussed by da Silva et al. and by studies focused on stingless bee honey quality, low sucrose content is generally associated with adequate enzymatic hydrolysis and absence of adulteration [30,34]. Nonetheless, the slightly higher sucrose values found in some spring racking samples may suggest incomplete inversion or early harvest of specific pots, especially under minimal-handling collection conditions. This is plausible in artisanal systems, where hive structure and access can constrain standardized harvesting.

The bioactive profile was the parameter set most clearly affected by seasonality. Total phenolic compounds, flavonoids, and antioxidant activity were consistently higher in spring samples, indicating that floral diversity during the blooming season enhances the incorporation of secondary metabolites into honey. This agrees with previous studies emphasizing the strong relationship between pollen/floral origin and antioxidant potential in stingless bee honey [3,15,39,44].

A similar seasonal pattern was observed by J.A. Limpias-Hurtado et al., who reported higher concentrations of bioactive compounds in spring-harvested *A. mellifera* honeys from Santa Cruz, Bolivia, further supporting the role of surrounding vegetation and flowering dynamics as key drivers of antioxidant potential in regional honeys [41].

Ávila et al. demonstrated that botanical origin is a major determinant of the biological properties of Brazilian stingless bee honeys [3], while Ranneh et al. reported strong links between phenolic profile and antioxidant performance in Malaysian stingless bee honey [39]. In the present study, the association between lower L* values and higher phenolic content also aligns with the literature, indicating that darker honeys generally contain higher concentrations of antioxidant compounds [28,45]. Therefore, the seasonal enrichment in phenolics observed here is not merely a compositional curiosity, but a strong indicator of enhanced functional value.

The antioxidant assays further support this interpretation. ORAC and ABTS^+^ were particularly responsive to the phenolic differences among samples, whereas DPPH showed a more uniform pattern, which is not unusual given the distinct mechanisms and sensitivities of antioxidant assays. Escriche et al. discussed that antioxidant capacity methods do not always respond equally to the same phenolic matrix [36], and Negera et al. similarly observed method-dependent differences in stingless bee honey antioxidant characterization [37]. Thus, the use of multiple assays in the present study strengthens the robustness of the antioxidant assessment. The higher antioxidant performance of spring samples suggests that ecological conditions, and especially floral diversity, are stronger drivers of functional quality than harvesting method alone.

This interpretation is reinforced by the PCA. Rather than simply separating samples visually, the PCA integrates the relationships among physicochemical and biological variables and suggests that honey variability may be structured along at least two major axes: a bioactive-functional axis dominated by phenolics, protein, and ORAC, and a second axis linked to antioxidant reactivity and selected physicochemical parameters. The tighter clustering of optimized samples suggests that improved harvesting practices may reduce dispersion and improve compositional consistency, even if they do not always substantially alter absolute values. These observations may be particularly relevant from a quality-control perspective. In small-scale meliponiculture systems, reducing compositional heterogeneity may be more relevant than modifying composition itself, since standardization is essential for market access, quality certification, and consumer trust. By contrast, the greater dispersion observed in conventional and racking samples may indicate that environmental exposure, manual handling, and nest-specific factors contribute to broader physicochemical heterogeneity.

Taken together, these findings suggest that *T. fiebrigi* honey quality in Bolivia is governed by an interaction among ecological drivers, colony-level variability, and harvesting conditions. Sesonality primarily modulates the availability of floral resources and, therefore, the nutritional and bioactive composition of the honey, whereas optimized harvesting appears to contribute mainly to consistency and process reliability. This distinction may be highly relevant for Bolivian meliponiculture: while producers may not be able to control climatic and botanical variability, they can improve product homogeneity and commercial quality through better harvesting practices. In that sense, the present study not only contributes new data on an understudied native honey but also offers a practical framework for future standardization and valorization efforts in Bolivia.

### Future Research and Perspectives

Although this study provides foundational data for Bolivian T. fiebrigi honey, several aspects warrant further investigation. First, a broader seasonal sampling covering multiple annual cycles would allow differentiation between climatic anomalies and recurrent ecological patterns. Second, detailed metabolomic profiling, including trehalulose quantification and untargeted LC–MS analysis, would deepen understanding of species-specific bioactive signatures [46]. Third, controlled storage studies could clarify the stability of phenolic compounds and antioxidant capacity under different post-harvest conditions. Finally, microbiological characterization of pot-associated microbial communities may elucidate their role in honey maturation and preservation mechanisms.

Developing region-specific physicochemical benchmarks adapted to Bolivia’s tropical dry forest ecosystem will be essential for regulatory recognition and international commercialization of native stingless bee honey.

This need is further supported by the findings of J.A. Limpias-Hurtado et al., who emphasized the importance of integrating melissopalynological analysis with advanced chromatographic techniques to establish robust botanical and functional markers for Bolivian honeys. Such approaches would enhance traceability, regional valorization, and potential nutraceutical certification.

## 4. Materials and Methods

### 4.1. Honey Samples

Honey samples (≈130 g per hive) were collected from 11 *Tetragonisca fiebrigi* colonies located at the Centro de Estudios del Bosque Seco Tropical Alta Vista (CEBST), situated 19 km northeast of Concepción, Santa Cruz, Bolivia (16°05′51.7″ S, 61°53′28.5″ W). Of these, nine samples were obtained from artificial hives and two from natural nests. Sampling was performed manually during two seasons—winter (23 May 2023) and spring (11 October 2023)—between 7:00 and 9:00 a.m., avoiding the use of smoke or chemicals to minimize colony disturbance. Environmental parameters were recorded at the time of collection, including relative humidity, temperature, heat index, and date of last rainfall (Table 3). These environmental parameters, particularly humidity and temperature, were considered relevant for interpreting the physicochemical variations among samples collected in different seasons.

#### Harvesting Methods and Sample Coding

Three distinct honey harvesting methods were employed:

- Conventional method (C): Based on traditional practices used at CEBST, involving basic hygiene and the use of clean, sterilized containers.
- Optimized method (O): Developed according to Codex Alimentarius hygiene standards, with stricter sanitary measures and the use of sterile, hermetically sealed materials [47].
- Racking method (T): Direct decantation from natural nests with minimal intervention, involving filtration to remove solid particles (wood, sand, etc.).

Samples were coded to reflect the season and harvesting method: P = Spring, I = Winter, C = Conventional, O = Optimized, T = Racking. For example, “IC1” denotes a winter sample collected using the conventional method from colony 1.

During winter, seven samples were collected: four using the optimized method (IO1–IO4) and three using the conventional method (IC1–IC3). One optimized sample (IO2) was noted for containing leaf material within the comb. In spring, four samples were obtained: one by the conventional method (PC1), one by the optimized method (PO1), and two by the racking method (PT1, PT2), due to natural nest accessibility.

Although racking samples involved direct extraction, they were initially collected using conventional tools and later strained to ensure sample purity. Additionally, IC3 was obtained by manually squeezing leftover comb material from IO3 and was categorized under the conventional method due to the post-harvest manipulation involved.

Due to limited honey production per colony and logistical constraints on nest accessibility, it was not possible to collect large volumes or obtain duplicate samples from each hive. This restriction was considered during experimental planning and statistical analysis.

### 4.2. Physicochemical Characterization

The physicochemical properties of honey were determined following the protocols established by the Association of Official Analytical Chemists (1990) and the International Honey Commission (2009) [48,49].

#### 4.2.1. pH

The pH of the honey samples was determined in triplicate following the methodology proposed by the International Honey Commission (IHC) [48]. Each solution was prepared by dissolving 10 g of honey in 75 milliliters of distilled water. The pH of each solution was then measured using a properly calibrated Hanna HI2210 pH meter (Hanna Instruments, Johannesburg, South Africa).

#### 4.2.2. Moisture Content

Moisture content (%) was determined gravimetrically [48]. Approximately 5 g of honey was weighed and dried in a convection oven at 105 °C for 48 h until constant weight. Moisture percentage was calculated from weight loss after drying using the following equation:

% Moisture = [(initial weight − dry weight)/initial weight] × 100(1)

Results were expressed as percentage (%) of honey moisture content.

#### 4.2.3. Ash Content

The ash content was determined in triplicate by calcination in a muffle furnace, following the methodology of the International Honey Commission (IHC) with minor modifications [48]. Five grams of honey were calcinated at 200 °C on a magnetic hot plate and subsequently incinerated at 550 °C for 6 h. After cooling, the residual ash was weighed using an analytical balance. The ash content was expressed as grams per 100 g.

#### 4.2.4. Protein Content

Total protein content was determined in duplicate using the Kjeldahl method (AOAC 960.52) [50]. Approximately 1 mL of honey sample diluted at a 1:10 ratio was digested with sulfuric acid in the presence of 0.3% CuSO_4_·5H_2_O as a catalyst until complete mineralization was achieved. The digested samples were subsequently neutralized and distilled, and the released ammonia was quantified by titration. Protein content was calculated using a nitrogen-to-protein conversion factor of 6.25 and expressed as g of protein/100 g of honey [51].

#### 4.2.5. Water Activity

Water activity (Aw) was measured in pure honey using a HygroLab water activity meter (Rotronic AG, Bassersdorf, Switzerland). This parameter reflects the amount of unbound water available for microbial growth and chemical reactions and is critical for evaluating honey’s stability and shelf life.

#### 4.2.6. Color Analysis

Honey color was assessed using a Minolta CR-400 colorimeter (Minolta Co, Ltd., Osaka, Japan) that measures CIELab variables: L* (lightness), a* (red-green), and b* (yellow-blue). Approximately 3 g of honey were placed in a Petri dish, and color measurements were recorded [14,52]. The apparatus was duly calibrated using a white reference tile, and measurements were conducted at ten distinct points on each honey sample, with the resultant mean values being recorded for each sample.

#### 4.2.7. Total and Reduced Sugars

Total and reducing sugars were quantified by a Beckman Coulter System Gold HPLC (Knauer, Berlin, Germany) coupled to RI detectors, and equipped with an Aminex 37-H column (Bio-Rad, Berkeley, USA)., following the International Honey Commission (IHC) methodology with minor modifications [53]. Honey samples were prepared by dissolving 5 g of honey in 40 mL of water, homogenized, and filtered prior to analysis. Quantification of fructose, glucose, and sucrose was performed using external calibration curves prepared from analytical-grade standards at concentrations ranging from approximately 0.06 to 2.12 mg/mL. All analyses were carried out in triplicate, including sample preparation and chromatographic analysis, to ensure analytical reproducibility. Sugar contents were expressed as grams per 100 g of honey.

### 4.3. Biological Properties

#### 4.3.1. Total Phenolic Content (TPC)

The total phenolic content of honey samples was determined using the Folin–Ciocalteu method, based on the protocol described by Coscueta et al. (2018) [54], with minor modifications. For each measurement, 100 µL of honey was diluted in 900 µL of pure methanol, filtered, and 30 µL of the resulting solution was transferred to a 96-well microplate. Subsequently, 100 µL of Folin–Ciocalteu reagent and 100 µL of sodium carbonate anhydride solution were added. The mixture was incubated for 30 min at 25 °C, and the absorbance was measured at 760 nm using a multimode microplate reader.

Methanol was used as a blank. A standard calibration curve was constructed using gallic acid solutions (0.025–200 mg/L). Results were expressed as grams of gallic acid equivalents per 100 g of honey (mg GAE/100 g honey). Analyses were performed in triplicate.

#### 4.3.2. Total Flavonoid Content

The total flavonoid content of honey samples was determined following the method described by Meda et al. (2005) [55], with minor modifications. Honey samples were diluted in methanol to obtain a 0.25% (*m*/*v*) solution. Then, 1 mL of the diluted sample was mixed with 75 µL of 5% sodium nitrite (NaNO_2_) and allowed to react for 5 min. Next, 150 µL of 10% aluminum chloride hexahydrate (AlCl_3_·6H_2_O) was added, and the mixture was incubated for 6 min. Finally, 500 µL of 1 M sodium hydroxide (NaOH) was added, and the volume was adjusted to 2.5 mL with distilled water.

The absorbance of the resulting, red-colored complex was measured at 510 nm using a spectrophotometer, with methanol as the blank. A standard calibration curve was constructed using quercetin solutions (0–100 mg/L). The results were expressed as grams of quercetin equivalents per 100 g of honey (mg QE/100 g honey). All measurements were performed in triplicate.

### 4.4. Antioxidant Activity Assays

#### 4.4.1. DPPH (2,2-Diphenyl-1-Picrylhydrazyl) Free Radical-Scavenging Assay

The antioxidant activity of honey samples was evaluated using the DPPH (2,2-diphenyl-1-picrylhydrazyl) radical-scavenging assay, following the method described by Brand-Williams et al. (1995) with slight modifications [56,57].

Honey samples were diluted to a 10% (*w*/*v*) solution in methanol. Subsequently, 25 µL of the diluted sample was mixed with 175 µL of a freshly prepared DPPH• working solution (0.06 mM in methanol) in a 96-well microplate. The mixture was incubated in the dark at 25 °C for 30 min, and the absorbance was measured at 515 nm using a multimode microplate reader (Synergy H1, BioTek, Winooski, VT, USA).

A Trolox calibration curve was constructed using standard solutions ranging from 25 µM to 250 µM. The radical scavenging activity was expressed as moles of Trolox equivalents per 100 g of honey (mol TE/100 g honey). All measurements were performed in triplicate.

#### 4.4.2. ABTS+ (2,2’-Azino-Bis (3-Ethylbenzothiazoline-6-Sulfonic Acid))

The antioxidant capacity of honey samples was also assessed using the ABTS^+^ (2,2′-azino-bis (3-ethylbenzothiazoline-6-sulfonic acid)) radical cation assay, following the protocol described by Gonçalves et al. (2009) with minor modifications [58,59]. Honey samples were diluted to a 20% (*w*/*v*) solution in distilled water. For each measurement, 20 µL of the diluted sample was mixed with 180 µL of the ABTS^+^ working solution in a 96-well microplate. The mixture was incubated at 30 °C for 5 min, and absorbance was read at 734 nm using a multimode plate reader (Synergy H1, BioTek, Winooski, VT, USA).

A Trolox standard curve was prepared using concentrations ranging from 25 µM to 250 µM in methanol. Results were expressed as moles of Trolox equivalents per 100 g of honey (mol TE/100 g honey). All measurements were conducted in triplicate.

#### 4.4.3. ORAC (Oxygen Radical Absorbance Capacity)

The antioxidant capacity of honey samples was further evaluated using the ORAC (Oxygen Radical Absorbance Capacity) assay, based on the method described by Dávalos, Gómez-Cordovés, and Bartolomé (2004) with slight modifications [54,60,61].

A 1% (*w*/*v*) honey solution was prepared in phosphate buffer (pH 7.4). The assay was conducted in 96-well black microplates. After pre-incubating the samples and Trolox standards with the fluorescent probe, the reaction was initiated by adding a 12 mM AAPH (2,2′-azobis(2-amidinopropane) dihydrochloride) solution.

Fluorescence readings (excitation at 485 nm and emission at 520 nm) were recorded every minute for 140 min using a multimode plate reader (Synergy H1, BioTek, Winooski, VT, USA). Antioxidant capacity was calculated based on the Net Area Under the Curve (AUC) and expressed as moles of Trolox equivalents per 100 g of honey (mol TE/100 g honey).

All measurements were performed in triplicate to ensure precision and reproducibility.

### 4.5. Statistical Analysis

Physicochemical parameters and bioactive compound data were analyzed using one-way analysis of variance (ANOVA) with a 95% confidence interval to assess differences among samples according to extraction method and season (winter and spring). When significant differences were detected, Tukey’s post-hoc test was applied for multiple comparisons using a significance level set at *p* < 0.05. Significant differences among means are indicated by different letters in the tables.

Additionally, a Principal Component Analysis (PCA) was performed to explore correlation patterns among variables using Minitab^®^ software (version 17.1.0, LEAD Technologies, Inc., Charlotte, NC, USA). Variables were analyzed using their original scales, as the PCA was intended as an exploratory multivariate assessment of the relationships among physicochemical and biological parameters. Detailed PCA outputs, including explained variance and variable contributions, are provided in the Supplementary Material.

## 5. Conclusions

This study evaluated the impact of different collecting methods and seasonal variation on the physicochemical and antioxidant properties of *Tetragonisca fiebrigi* honey from the tropical dry forests of Bolivia. Honey collected during spring exhibited higher concentrations of phenolic compounds, flavonoids, and greater antioxidant capacity compared to winter samples, likely due to increased floral availability and nectar diversity during the blooming season. Among the evaluated samples, honey obtained using the optimized collecting method showed the highest overall quality, characterized by greater physicochemical stability, better preservation of bioactive compounds, and lower variability among samples.

The results indicate that both seasonal variation and harvesting practices significantly influence honey quality. Optimized collection procedures contributed to more stable pH values, improved preservation of antioxidant compounds, and greater homogeneity in physicochemical characteristics. Principal component analysis further supported these findings by showing tighter clustering patterns among samples collected using the optimized method.

Importantly, this research represents the first scientific study to characterize the physicochemical and antioxidant properties of stingless bee honey from Bolivia’s tropical dry forests. These findings provide valuable baseline information for the development of quality standards, sustainable harvesting protocols, and conservation strategies adapted to the ecological conditions of the region.

Overall, the study highlights the potential of T. fiebrigi honey as a functional food with significant nutritional and antioxidant value. The implementation of optimized harvesting practices, together with consideration of seasonal influences, may improve product quality, promote market valorization, and strengthen sustainable meliponiculture in Bolivia while contributing to biodiversity conservation and rural development.

## Figures and Tables

**Table 1:** Physicochemical compounds of native bee honey expressed in g/100 g.

	IC1	IC2	IC3	IO1	IO2	IO3	IO4	PC1	PO1	PT1	PT2
pH	5.13 ± 0.03 ^DE^	4.94 ± 0.03 ^F^	4.59 ± 0.04 ^I^	5.14 ± 0.01 ^D^	4.84 ± 0.02 ^G^	4.93 ± 0.03 ^F^	4.73 ± 0.03 ^H^	5.07 ± 0.04 ^E^	5.39 ± 0.01 ^C^	6.04 ± 0.01 ^B^	6.15 ± 0.01 ^A^
Ash Content	12.48 ± 1.08 ^A^	12.53 ± 0.23^A^	NR*	11.96 ± 0.39 ^A^	11.73 ± 0.48 ^A^	12.47 ± 0.35 ^A^	12.38 ± 0.52 ^A^	12.40 ± 1.08 ^A^	12.04 ± 0.42 ^A^	12.34 ± 0.46 ^A^	11.71 ± 0.28 ^A^
Moisture ^a^	30.50 ± 0.71 ^A^	31.00 ± 0.00 ^A^	NR*	30.00 ± 0.00 ^A^	44.00 ± 14.10 ^A^	32.00 ± 0.00 ^A^	30.00 ± 0.00 ^A^	31.00 ± 0.00^A^	31.00 ± 0.00 ^A^	27.50 ± 6.36 ^A^	34.00 ± 0.00 ^A^
Protein	10.65 ± 0.62 ^ABCD^	10.12 ± 0.81 ^BCD^	8.93 ± 2.35 ^CD^	1.58 ± 3.78 ^E^	1.32 ± 1.55 ^E^	7.18 ± 0.50 ^DE^	13.10 ± 0.00 ^ABCD^	12.65 ± 0.62 ^ABCD^	14.50 ± 0.12 ^ABC^	16.35 ± 0.56 ^A^	16.20 ± 1.55 ^AB^
Water Activity	0.72 ± 0.03 ^A^	0.73 ± 0.03 ^A^	NR*	0.70 ± 0.03 ^A^	0.74 ± 0.03 ^A^	0.73 ± 0.03 ^A^	0.75 ± 0.03 ^A^	0.70 ± 0.03 ^A^	0.71 ± 0.04 ^A^	0.70 ± 0.03 ^A^	0.69 ± 0.02 ^A^
Color (L*)	65.05 ± 0.53 ^DE^	64.14 ± 0.19 ^E^	67.48 ± 0.15 ^BC^	69.02 ± 0.07 ^A^	66.98 ± 0.30 ^C^	68.61 ± 0.54 ^AB^	65.43 ± 0.42 ^D^	59.76 ± 0.23 ^F^	58.18 ± 0.17 ^G^	65.51 ± 1.41 ^D^	58.06 ± 0.59 ^G^
Color (a*)	2.93 ± 0.04 ^A^	2.40 ± 0.03 ^D^	2.30 ± 0.01 ^E^	2.63 ± 0.02 ^B^	2.45 ± 0.03 ^CD^	2.61 ± 0.04 ^B^	2.37 ± 0.02 ^DE^	2.68 ± 0.06 ^B^	2.91 ± 0.02 ^A^	2.50 ± 0.05 ^C^	2.20 ± 0.05 ^F^
Color (b*)	8.91 ± 0.14 ^E^	9.07 ± 0.09 ^E^	6.82 ± 0.04 ^F^	5.17 ± 0.03 ^H^	5.15 ± 0.12 ^H^	4.70 ± 0.09 ^I^	5.91 ± 0.11 ^G^	12.58 ± 0.07 ^B^	12.99 ± 0.11 ^A^	10.92 ± 0.36 ^C^	10.25 ± 0.23 ^D^
Glucose	12.65 ± 1.10 ^E^	24.97 ± 1.38 ^AB^	23.65 ± 0.98 ^BC^	18.43 ± 1.15 ^CD^	10.60 ± 0.98 ^E^	13.88 ± 0.89 ^DE^	22.34 ± 0.21 ^BC^	25.72 ± 1.41 ^AB^	29.03 ± 2.12 ^A^	22.42 ± 2.21 ^BC^	15.83 ± 1.01 ^DE^
Sucrose	0.70 ± 0.48 ^D^	1.79 ± 0.03 ^BCD^	1.77 ± 0.11 ^BCD^	1.08 ± 0.10 ^CD^	0.79 ± 0.07 ^D^	1.25 ± 0.05 ^BCD^	1.31 ± 0.08 ^BCD^	2.36 ± 0.18 ^AB^	3.23 ± 0.29 ^A^	1.96 ± 0.73 ^BC^	1.63 ± 0.12 ^BCD^
Fructose	11.03 ± 1.14 ^B^	21.97 ± 1.14 ^A^	21.49 ± 0.94 ^A^	18.43 ± 1.08 ^A^	9.01 ± 0.67 ^B^	11.68 ± 1.02 ^B^	21.24 ± 0.22 ^A^	19.58 ± 0.96 ^A^	21.02 ± 1.82 ^A^	18.71 ± 0.61 ^A^	12.60 ± 1.09 ^B^
TPC ^b^	4.37 ± 0.21 ^CD^	3.83 ± 0.12 ^DE^	5.17 ± 0.25 ^C^	4.23 ± 0.21 ^CD^	4.13 ± 0.21 ^D^	3.17 ± 0.15 ^E^	3.71 ± 0.26 ^DE^	9.27 ± 0.47 ^B^	9.11 ± 0.44 ^B^	9.03 ± 0.59 ^B^	11.03 ± 0.32 ^A^
Flavonoids ^c^	10.47 ± 0.10 ^B^	10.43 ± 0.33 ^B^	13.64 ± 1.61 ^AB^	11.10 ± 0.24 ^AB^	10.70 ± 0.13 ^B^	10.21 ± 0.50 ^B^	23.08 ± 12.23 ^A^	12.48 ± 0.24 ^AB^	13.86 ± 0.89 ^AB^	14.65 ± 3.04 ^AB^	13.68 ± 2.56 ^AB^

^a^ % of Moisture per 100 g of honey; ^b^ TPC, total phenolics content, mg of GAE/100 g of honey; ^c^ mg of EQ/100 g of honey. Average values of replicates performed for each assay (3n) ± standard deviations, except for color parameters (10n) ± standard deviations and for protein, ash, and moisture parameters (2n) ± standard deviations. L* = Luminosity, a* = red/green coordinates, b* = yellow/blue coordinates. NR* = Tests not performed. Different letters (A, B, C, D, E, F, G, H, I) in the same row indicate significant differences between honey samples overall according to Tukey’s test (*p* < 0.05). Samples were coded to reflect the season and harvesting method: P = Spring, I = Winter, C = Conventional, O = Optimized, T = Racking. For example, “IC1” denotes a winter sample collected using the conventional method from colony 1.

**Table 2:** Antioxidant activity in native bee honey.

	IC1	IC2	IC3	IO1	IO2	IO3	IO4	PC1	PO1	PT1	PT2
ABTS+ (mol TE/100 g of honey)	2.76 ± 0.10 ^D^	2.60 ± 0.03 ^D^	6.96 ± 0.11 ^A^	3.64 ± 0.12 ^C^	4.42 ± 0.12 ^B^	3.98 ± 0.28 ^C^	3.63 ± 0.16 ^C^	2.90 ± 0.14 ^D^	1.41 ± 0.05 ^E^	ND *	6.60 ± 0.16 ^A^
*DPPH* (mol TE/100 g of honey)	0.66 ± 0.22 ^A^	0.51 ± 0.30 ^A^	0.66 ± 0.24 ^A^	0.46 ± 0.25 ^A^	0.53 ± 0.19 ^A^	0.86 ± 0.11 ^A^	0.57 ± 0.11 ^A^	0.64 ± 0.01 ^A^	0.32 ± 0.18 ^A^	1.14 ± 0.80 ^A^	1.33 ± 0.27 ^A^
*ORAC* (mol TE/100 g of honey)	10.96 ± 0.42 ^CD^	10.33 ± 0.51 ^CDE^	13.36 ± 0.92 ^C^	6.63 ± 2.29 ^E^	10.70 ± 0.27 ^CD^	8.06 ± 1.61 ^DE^	8.87 ± 2.20 ^DE^	22.94 ± 1.55 ^AB^	25.93 ± 0.32 ^A^	25.11 ± 1.79 ^AB^	21.67 ± 0.13 ^B^

Average values of the replicates performed for each test (3n) ± standard deviations. ND * = values not detected. Different letters (A, B, C, D, E) in the same row indicate significant differences between honeys overall according to Tukey’s test (*p* < 0.05). Samples were coded to reflect the season and harvesting method: P = Spring, I = Winter, C = Conventional, O = Optimized, T = Racking. For example, “IC1” denotes a winter sample collected using the conventional method from colony 1.

**Table 3:** Environmental parameters recorded during honey sampling events.

Samples		Date of Collection	Relative Humidity (%)	Date of the Last Rain	Daily Temperature (°C)	Heat Index (°C)
IO1 IO2 IO3 IO4	IC1 IC2 IC3	23/05/2023	89.35	05/05/2023	24.58	29.36
PO1 PT1	PC1 PT2	11/10/2023	66.09	30/09/2023	30.76	37.46

Samples were coded to reflect the season and harvesting method: P = Spring, I = Winter, C = Conventional, O = Optimized, T = Racking. For example, “IC1” denotes a winter sample collected using the conventional method from colony 1.

## Data Availability

The original contributions presented in this study are included in the article/Supplementary Material. Further inquiries can be directed to the corresponding author.

## Supplementary Materials

The following supporting information can be downloaded at: https://www.mdpi.com/article/10.3390/molecules31111819/s1, The One-way ANOVA and the Principal Component Analysis for all the samples: IC1; IC2; IC3; PT1; PT2; IO1; IO2; IO3; IO4; PO1; PC1 are presented in the Supplementary Materials.

## Abbreviations

The following abbreviations are used in this manuscript:

ABTS	2,2′-azino-bis (3-ethylbenzothiazoline-6-sulfonic acid)
AOAC	Association of Official Analytical Chemists
AAPH	2,2′-azobis(2-amidinopropane) dihydrochloride
AUC	Area Under the Curve
Aw	Water activity
CEBST	Centro de Estudios del Bosque Seco Tropical Alta Vista
DPPH	2,2-diphenyl-1-picrylhydrazyl
GAE	Gallic acid equivalents
HPLC	High-performance liquid chromatography
IHC	International Honey Commission
LC–MS	Liquid chromatography–mass spectrometry
ND	Not detected
NR	Not reported/Not performed
ORAC	Oxygen Radical Absorbance Capacity
PCA	Principal Component Analysis
QE	Quercetin equivalents
SBH	Stingless bee honey
TE	Trolox equivalents
TPC	Total phenolic content

## References

[1] Menezes C., Vollet-Neto A., Contrera F.A.F.L., Venturieri G.C., Imperatriz-Fonseca V.L. (2013). The Role of Useful Microorganisms to Stingless Bees and Stingless Beekeeping. Pot-Honey.

[2] Bradbear N. (2009). Bees and their role in forest livelihoods. Non-Wood Forest Products.

[3] Ávila S., Hornung P.S., Teixeira G.L., Malunga L.N., Apea-Bah F.B., Beux M.R., Beta T., Ribani R.H. (2019). Bioactive compounds and biological properties of Brazilian stingless bee honey have a strong relationship with the pollen floral origin. Food Res. Int..

[4] Pimentel T.C., Rosset M., Sousa J.M.B., Oliveira L.I.G., Mafaldo I.M., Pintado M.M.E., Souza E.L., Magnani M. (2022). Stingless bee honey: An overview of health benefits and main market challenges. J. Food Biochem..

[5] Shamsudin S., Selamat J., Sanny M., Razak S.-B.A., Jambari N.N., Mian Z., Khatib A. (2019). Influence of origins and bee species on physicochemical, antioxidant properties and botanical discrimination of stingless bee honey. Int. J. Food Prop..

[6] Adler M., Martinez-Ugarteche M.T., Toledo M. (2022). Characteristics of nests of stingless bees (Apoidea: Meliponini) in the Lomerío (Santa Cruz) and Sirionó (Beni) territories, Bolivia. Kempffiana.

[7] Schvezov N., Pucciarelli A.B., Valdes B., Dallagnol A.M. (2020). Characterization of yateí (Tetragonisca fiebrigi) honey and preservation treatments: Dehumidification, pasteurization and refrigeration. Food Control.

[8] Campos J.F., dos Santos U.P., Rocha P.d.S.d., Damião M.J., Balestieri J.B.P., Cardoso C.A.L., Paredes-Gamero E.J., Estevinho L.M., Souza K.d.P., dos Santos E.L. (2015). Antimicrobial, Antioxidant, Anti-Inflammatory, and Cytotoxic Activities of Propolis from the Stingless Bee *Tetragonisca fiebrigi* (Jataí). Evid.-Based Complement. Altern. Med..

[9] Vit P., Gutiérrez M.G., Rodriguez-Malaver A.J., Aguilera G., Fernández-Díaz C., Tricio A.E. (2009). Comparison of honeys produced by the bee yateí (Tetragonisca fiebrigi) in Argentina and Paraguay. Acta Bioquím. Clín. Latinoam..

[10] Saravia-Nava A., Niemeyer H.M., Pinto C.F. (2018). Pollen Types Used by the Native Stingless Bee, Tetragonisca angustula (Latreille), in an Amazon-Chiquitano Transitional Forest of Bolivia. Neotrop. Entomol..

[11] Julika W.N., Ajit A., Ismail N., Aqilah N., Naila A., Sulaiman A.Z. (2020). Sugar profile and enzymatic analysis of stingless bee honey collected from local market in Malaysia. IOP Conf. Ser. Mater. Sci. Eng..

[12] Bijlsma L., de Bruijn L.L.M., Martens E.P., Sommeijer M.J. (2006). Water content of stingless bee honeys (*Apidae, Meliponini*): Interspecific variation and comparison with honey of *Apis mellifera*. Apidologie.

[13] Ikhsan L.N., Chin K.-Y., Ahmad F. (2022). Methods of the Dehydration Process and Its Effect on the Physicochemical Properties of Stingless Bee Honey: A Review. Molecules.

[14] Esa N.E.F., Ansari M.N.M., Razak S.I.A., Ismail N.I., Jusoh N., Zawawi N.A., Jamaludin M.I., Sagadevan S., Nayan N.H.M. (2022). A Review on Recent Progress of Stingless Bee Honey and Its Hydrogel-Based Compound for Wound Care Management. Molecules.

[15] Santos A.C.D., Biluca F.C., Braghini F., Gonzaga L.V., Costa A.C.O., Fett R. (2021). Phenolic composition and biological activities of stingless bee honey: An overview based on its aglycone and glycoside compounds. Food Res. Int..

[16] Chuttong B., Chanbang Y., Sringarm K., Burgett M. (2016). Physicochemical profiles of stingless bee (Apidae: Meliponini) honey from South East Asia (Thailand). Food Chem..

[17] Ávila S., Beux M.R., Ribani R.H., Zambiazi R.C. (2018). Stingless bee honey: Quality parameters, bioactive compounds, health-promotion properties and modification detection strategies. Trends Food Sci. Technol..

[18] Sujanto I.S.R., Ramly N.S., Ghani A.A., Huat J.T.Y., Alias N., Ngah N. (2021). The Composition and Functional Properties of Stingless Bee Honey: A Review. Malays. J. Appl. Sci..

[19] Lemos M.S., Venturieri G.C., Filho H.A.D., Dantas K.G.F. (2018). Evaluation of the physicochemical parameters and inorganic constituents of honeys from the Amazon region. J. Apic. Res..

[20] Bakar M.F.A., Sanusi S.B., Bakar F.I.A., Cong O.J., Mian Z. (2017). Physicochemical and Antioxidant Potential of Raw Unprocessed Honey From Malaysian Stingless Bees. Pak. J. Nutr..

[21] Braghini F., Gonzaga L.V., Costa A.C.O., Fett R. (2016). Physicochemical profiles, minerals and bioactive compounds of stingless bee honey (Meliponinae). J. Food Compos. Anal..

[22] Ismail N., Maulidiani M., Omar S., Zulkifli M.F., Radzi M.N.F.M., Ismail N., Jusoh A.Z., Roowi S., Yew W.M., Rudiyanto R. (2021). Classification of stingless bee honey based on species, dehumidification process and geographical origins using physicochemical and ATR-FTIR chemometric approach. J. Food Compos. Anal..

[23] deAlmeida-Muradian L.B., Stramm K.M., Horita A., Barth O.M., daSilva de Freitas A., Estevinho L.M. (2013). Comparative study of the physicochemical and palynological characteristics of honey from *Melipona subnitida* and *Apis mellifera*. Int. J. Food Sci. Technol..

[24] Nordin A., Sainik N.Q.A.V., Chowdhury S.R., Saim A.B., Idrus R.B.H. (2018). Physicochemical properties of stingless bee honey from around the globe: A comprehensive review. J. Food Compos. Anal..

[25] de Sousa J.M.B., de Souza E.L., Marques G., de Toledo Benassi M., Gullón B., Pintado M.M., Magnani M. (2016). Sugar profile, physicochemical and sensory aspects of monofloral honeys produced by different stingless bee species in Brazilian semi-arid region. LWT-Food Sci. Technol..

[26] Chirife J., Zamora M.C., Motto A. (2006). The correlation between water activity and % moisture in honey: Fundamental aspects and application to Argentine honeys. J. Food Eng..

[27] Zamora M.C., Chirife J., Roldán D. (2006). On the nature of the relationship between water activity and % moisture in honey. Food Control.

[28] Blasa M., Candiracci M., Accorsi A., Piacentini M.P., Albertini M.C., Piatti E. (2006). Raw Millefiori honey is packed full of antioxidants. Food Chem..

[29] Muhammad N.I.I., Sarbon N.M. (2023). Physicochemical profile, antioxidant activity and mineral contents of honey from stingless bee and honey bee species. J. Apic. Res..

[30] Agussalim A., Sabir A., Sahlan M., Agus A. (2023). Evaluation of stingless bee honey quality (Tetragonula laeviceps) based on their physicochemical from different origins. Biodiversitas J. Biol. Divers..

[31] Biluca F.C., da Silva B., Caon T., Mohr E.T.B., Vieira G.N., Gonzaga L.V., Vitali L., Micke G., Fett R., Dalmarco E.M. (2020). Investigation of phenolic compounds, antioxidant and anti-inflammatory activities in stingless bee honey (Meliponinae). Food Res. Int..

[32] Ja’afar N.L., Ab Aziz N.A., Zakaria Z., Mustapha M., Mohamed M., Mustafa M.Z., Hashim S. (2025). A narrative review on the physicochemical profiles, bioactive compounds, and therapeutic potentials of stingless bee honey. Discov. Food.

[33] Gadge A.S., Shirsat D.V., Soumia P.S., Pote C.L., Pushpalatha M., Pandit T.R., Dutta R., Kumar S., Ramesh S.V., Mahajan V. (2024). Physiochemical, biological, and therapeutic uses of stingless bee honey. Front. Sustain. Food Syst..

[34] da Silva P.M., Gauche C., Gonzaga L.V., Costa A.C.O., Fett R. (2016). Honey: Chemical composition, stability and authenticity. Food Chem..

[35] Escuredo O., Dobre I., Fernández-González M., Seijo M.C. (2014). Contribution of botanical origin and sugar composition of honeys on the crystallization phenomenon. Food Chem..

[36] Escriche I., Kadar M., Juan-Borrás M., Domenech E. (2014). Suitability of antioxidant capacity, flavonoids and phenolic acids for floral authentication of honey. Impact of industrial thermal treatment. Food Chem..

[37] Negera T., Degu A., Tigu F. (2024). Comparative analysis of the physicochemical, proximate, and antioxidant characteristics of stingless bee (*Meliponula beccarii*) honey from modern and wild beehives in Ethiopia. Food Sci. Nutr..

[38] da Silva Cruz L.F., Lemos P.V.F., Santos T.d.S., Tavares P.P.L.G., Nascimento R.Q., Almeida L.M.R., de Souza C.O., Druzian J.I. (2023). Storage conditions significantly influence the stability of stingless bee (*Melipona scutellaris)* honey. J. Apic. Res..

[39] Ranneh Y., Ali F., Zarei M., Akim A.M., Hamid H.A., Khazaai H. (2018). Malaysian stingless bee and Tualang honeys: A comparative characterization of total antioxidant capacity and phenolic profile using liquid chromatography-mass spectrometry. LWT-Food Sci. Technol..

[40] Vit P., Oddo L.P., Marano M.L., de Mejias E.S. (1998). Venezuelan stingless bee honeys characterized by multivariate analysis of physicochemical properties. Apidologie.

[41] Limpias-Hurtado J.A., Duran N.M., Giménez-Turba A., Nina N. (2026). Exploratory assessment of the chemical and antioxidant potential of Apis mellifera honeys from Santa Cruz, Bolivia. Food Chem. X.

[42] Sant’ana R.d.S., de Carvalho C.A.L., Oda-Souza M., Souza B.d.A., Dias F.d.S. (2020). Characterization of honey of stingless bees from the Brazilian semi-arid region. Food Chem..

[43] Anacleto D.d.A., Souza B.d.A., Marchini L.C., Moreti A.C.d.C.C. (2009). Composição de amostras de mel de abelha Jataí (Tetragonisca angustula latreille, 1811). Ciência Tecnol. Aliment..

[44] Mduda C.A., Hussein J.M., Muruke M.H. (2023). The effects of bee species and vegetation on the antioxidant properties of honeys produced by Afrotropical stingless bees (Hymenoptera, Apidae, Meliponini). J. Agric. Food Res..

[45] Bertoncelj J., Doberšek U., Jamnik M., Golob T. (2007). Evaluation of the phenolic content, antioxidant activity and colour of Slovenian honey. Food Chem..

[46] Santos M.M.D., Khan N., Lim L.Y., Locher C. (2024). Antioxidant Activity, Physicochemical and Sensory Properties of Stingless Bee Honey from Australia. Foods.

[47] Joint FAO/WHO Codex Alimentarius Commission (1992). Codex Alimentarius.

[48] AOAC (1990). Official Methods of Analytical Chemist.

[49] Bogdanov S. (2009). Harmonised Methods of the International Honey Commission. https://www.ihc-platform.net/ihcmethods2009.pdf.

[50] Boghossian M., Brassesco M.E., Miller F.A., Silva C.L., Brandão T.R. (2023). Thermosonication applied to kiwi peel: Impact on nutritional and microbiological indicators. Foods.

[51] ISO International Organization for Standardization. https://www.iso.org/.

[52] Gomes T., Feás X., Iglesias A., Estevinho L.M. (2011). Study of Organic Honey from the Northeast of Portugal. Molecules.

[53] Deng Q., Penner M.H., Zhao Y. (2011). Chemical composition of dietary fiber and polyphenols of five different varieties of wine grape pomace skins. Food Res. Int..

[54] Coscueta E.R., Malpiedi L.P., Nerli B.B. (2018). Micellar systems of aliphatic alcohol ethoxylates as a sustainable alternative to extract soybean isoflavones. Food Chem..

[55] Meda A., Lamien C.E., Romito M., Millogo J., Nacoulma O.G. (2005). Determination of the total phenolic, flavonoid and proline contents in Burkina Fasan honey, as well as their radical scavenging activity. Food Chem..

[56] Brand-Williams W., Cuvelier M.E., Berset C. (1995). Use of a free radical method to evaluate antioxidant activity. LWT-Food Sci. Technol..

[57] Schaich K.M., Tian X., Xie J. (2015). Reprint of “Hurdles and pitfalls in measuring antioxidant efficacy: A critical evaluation of ABTS, DPPH, and ORAC assays”. J. Funct. Foods.

[58] Gonçalves B., Falco V., Moutinho-Pereira J., Bacelar E., Peixoto F., Correia C. (2009). Effects of Elevated CO_2_ on Grapevine (*Vitis vinifera* L.): Volatile Composition, Phenolic Content, and in Vitro Antioxidant Activity of Red Wine. J. Agric. Food Chem..

[59] Sanchez-Moreno C. (2002). Review: Methods Used to Evaluate the Free Radical Scavenging Activity in Foods and Biological Systems. Food Sci. Technol. Int..

[60] Scripcă L.A., Norocel L., Amariei S. (2019). Comparison of Physicochemical, Microbiological Properties and Bioactive Compounds Content of Grassland Honey and other Floral Origin Honeys. Molecules.

[61] Mahani, Ahmed A.O.M., Nurhadi B. (2022). Evaluate antioxidant activity, phenolic content and colour of Indonesian stingless bee honey and sting bee honey cultivated in Indonesia. Asian J. Pharm. Clin. Res..

