# Supplementary material for: Influence of Harvesting and Seasonal Variability on the Physicochemical and Antioxidant Properties of Native Bee (Tetragonisca fiebrigi) Honey from Bolivia’s Tropical Dry Forests
Source: Molecules. 2026 May 25;31(11):1819. doi: 10.3390/molecules31111819 (PMC13258332; doi:10.3390/molecules31111819)
Supplement: Supplementary file 1 [file molecules-31-01819-s001.zip › molecules-4293621-supplementary.pdf]

Article

Influence of Harvesting and Seasonal Variability on the Physicochemical and Antioxidant Properties of Native Bee *(Tetragonisca fiebrigi)* Honey from Bolivia's Tropical Dry Forests

Alejandra Romero-Padilla [1], Luís M. G. Castro [2], Manuela Pintado [2,*] and María Emilia Brassesco[2]

[1] Biotechnology, Universidad Católica Boliviana "San Pablo". Santa Cruz, Bolivia; alejandra.romero2201@gmail.com
[2] Universidade Católica Portuguesa, CBQF - Centro de Biotecnologia e Química Fina – Laboratório Associado, Escola Superior de Biotecnologia, Rua Diogo Botelho 1327, 4169-005 Porto, Portugal; lgcastro@ucp.pt, mbrassesco@ucp.pt
* Correspondence: mpintado@ucp.pt

One-way ANOVA for all the samples: IC1; IC2; IC3; PT1; PT2; IO1; IO2; IO3; IO4; PO1; PC1.

The method was applied to all the parameters presented in the original manuscripts (Tables 1 and 2):

Null hypothesis	All means are equal
Alternative hypothesis	At least one mean is different
Significance level	α = 0.05

Equal variances were assumed for the analysis.

pH

Factor Information

Factor	Levels	Values
Factor	11	IC1; IC2; IC3; PT1; PT2; IO1; IO2; IO3; IO4; PO1; PC1

Analysis of Variance

Source	DF	Adj SS	Adj MS	F-Value	P-Value
Factor	10	7.56907	0.756907	1200.75	0.000
Error	22	0.01387	0.000630		
Total	32	7.58294			

Model Summary

S	R-sq	R-sq(adj)	R-sq(pred)
0.0251070	99.82%	99.73%	99.59%

Means

Factor	N	Mean	StDev	95% CI
IC1	3	5.1247	0.0250	(5.0946; 5.1547)
IC2	3	4.9377	0.0297	(4.9076; 4.9677)
IC3	3	4.5870	0.0393	(4.5569; 4.6171)
PT1	3	6.0397	0.0361	(6.0096; 6.0697)
PT2	3	6.14700	0.01044	(6.11694; 6.17706)
IO1	3	5.14300	0.00700	(5.11294; 5.17306)
IO2	3	4.8440	0.0208	(4.8139; 4.8741)

IO3	3	4.9253	0.0281	(4.8953; 4.9554)
IO4	3	4.7340	0.0305	(4.7039; 4.7641)
PO1	3	5.39133	0.01358	(5.36127; 5.42140)
PC1	3	5.06633	0.00902	(5.03627; 5.09640)

Pooled StDev = 0.0251070

Tukey Pairwise Comparisons

Grouping Information Using the Tukey Method and 95% Confidence

Factor	N	Mean	Grouping
PT2	3	6.14700	A
PT1	3	6.0397	B
PO1	3	5.39133	C
IO1	3	5.14300	D
IC1	3	5.1247	D E
PC1	3	5.06633	E
IC2	3	4.9377	F
IO3	3	4.9253	F
IO2	3	4.8440	G
IO4	3	4.7340	H
IC3	3	4.5870	I

Means that do not share a letter are significantly different.

ASH

Factor Information

Factor	Levels	Values
Factor	10	IC1; IC2; PT1; PT2; IO1; IO2; IO3; IO4; PO1; PC1

Analysis of Variance

Source	DF	Adj SS	Adj MS	F-Value	P-Value
Factor	9	1.787	0.1986	0.55	0.810
Error	10	3.617	0.3617		
Total	19	5.404			

Model Summary

S	R-sq	R-sq(adj)	R-sq(pred)
0.601425	33.07%	0.00%	0.00%

Means

Factor	N	Mean	StDev	95% CI
IC1	2	12.477	1.077	(11.530; 13.425)
IC2	2	12.532	0.232	(11.584; 13.480)
PT1	2	12.337	0.387	(11.390; 13.285)
PT2	2	11.709	0.481	(10.762; 12.657)
IO1	2	11.957	0.350	(11.009; 12.904)
IO2	2	11.733	0.518	(10.786; 12.681)
IO3	2	12.467	1.079	(11.520; 13.415)
IO4	2	12.384	0.423	(11.437; 13.332)
PO1	2	12.038	0.459	(11.090; 12.985)
PC1	2	12.395	0.277	(11.448; 13.343)

Pooled StDev = 0.601425

Tukey Pairwise Comparisons

Grouping Information Using the Tukey Method and 95% Confidence

Factor	N	Mean	Grouping
IC2	2	12.532	A
IC1	2	12.477	A
IO3	2	12.467	A
PC1	2	12.395	A
IO4	2	12.384	A
PT1	2	12.337	A
PO1	2	12.038	A
IO1	2	11.957	A
IO2	2	11.733	A
PT2	2	11.709	A

Means that do not share a letter are significantly different.

MOISTURE

Factor Information

Factor	Levels	Values
Factor	10	IC1; IC2; PT1; PT2; IO1; IO2; IO3; IO4; PO1; PC1

Analysis of Variance

Source	DF	Adj SS	Adj MS	F-Value	P-Value
Factor	9	0.03628	0.004031	1.67	0.217
Error	10	0.02410	0.002410		
Total	19	0.06038			

Model Summary

S	R-sq	R-sq(adj)	R-sq(pred)
0.0490918	60.09%	24.16%	0.00%

Means

Factor	N	Mean	StDev	95% CI
IC1	2	30.500	0.707	(0.22765; 0.38235)
IC2	2	31.00	0.0000	(0.2327; 0.3873)
PT1	2	27.50	6.36	(0.1977; 0.3523)
PT2	2	34.00	0.0000	(0.2627; 0.4173)
IO1	2	30.00	0.0000	(0.2227; 0.3773)
IO2	2	44.00	14.10	(0.363; 0.517)
IO3	2	32.00	0.0000	(0.2427; 0.3973)
IO4	2	30.00	0.0000	(0.2227; 0.3773)
PO1	2	31.00	0.0000	(0.2327; 0.3873)
PC1	2	31.00	0.0000	(0.2327; 0.3873)

Pooled StDev = 0.0490918

Tukey Pairwise Comparisons

Grouping Information Using the Tukey Method and 95% Confidence

Factor	N	Mean	Grouping
IO2	2	44.0	A
PT2	2	34.00	A
IO3	2	32.00	A
PC1	2	31.00	A
PO1	2	31.00	A
IC2	2	31.00	A
IC1	2	30.500	A
IO4	2	30.00	A
IO1	2	30.00	A
PT1	2	27.50	A

Means that do not share a letter are significantly different.

PROTEIN

Factor Information

Factor	Levels	Values
Factor	11	IC1; IC2; IC3; PT1; PT2; IO1; IO2; IO3; IO4; PO1; PC1

Analysis of Variance

Source	DF	Adj SS	Adj MS	F-Value	P-Value
Factor	10	541.95	54.195	22.71	0.000
Error	11	26.25	2.386		
Total	21	568.20			

Model Summary

S	R-sq	R-sq(adj)	R-sq(pred)
1.54479	95.38%	91.18%	81.52%

Means

Factor	N	Mean	StDev	95% CI
IC1	2	10.650	0.636	(8.246; 13.054)
IC2	2	10.120	0.820	(7.716; 12.524)
IC3	2	8.93	2.35	(6.53; 11.34)
PT1	2	16.35	3.75	(13.95; 18.75)
PT2	2	16.20	1.56	(13.80; 18.60)
IO1	2	1.575	0.615	(-0.829; 3.979)
IO2	2	1.3150	0.1202	(-1.0892; 3.7192)
IO3	2	7.180	0.495	(4.776; 9.584)
IO4	2	13.10	0.00	(10.70; 15.50)
PO1	2	14.500	0.566	(12.096; 16.904)
PC1	2	12.65	1.48	(10.25; 15.05)

Pooled StDev = 1.54479

Tukey Pairwise Comparisons

Grouping Information Using the Tukey Method and 95% Confidence

Factor	N	Mean	Grouping
PT1	2	16.35	A
PT2	2	16.20	A B
PO1	2	14.500	A B C

IO4	2	13.10	A B C D
PC1	2	12.65	A B C D
IC1	2	10.650	A B C D
IC2	2	10.120	B C D
IC3	2	8.93	C D
IO3	2	7.180	D E
IO1	2	1.575	E
IO2	2	1.3150	E

Means that do not share a letter are significantly different.

GLUCOSE

Factor Information

Factor	Levels	Values
Factor	11	IC1; IC2; IC3; PT1; PT2; IO1; IO2; IO3; IO4; PO1; PC1

Analysis of Variance

Source	DF	Adj SS	Adj MS	F-Value	P-Value
Factor	10	723.63	72.363	40.68	0.000
Error	11	19.57	1.779		
Total	21	743.20			

Model Summary

S	R-sq	R-sq(adj)	R-sq(pred)
1.33373	97.37%	94.97%	89.47%

Means

Factor	N	Mean	StDev	95% CI
IC1	2	12.654	1.096	(10.579; 14.730)
IC2	2	24.967	1.377	(22.892; 27.043)
IC3	2	23.648	0.980	(21.573; 25.724)
PT1	2	22.419	1.145	(20.343; 24.495)
PT2	2	15.826	0.983	(13.750; 17.902)
IO1	2	18.955	0.891	(16.879; 21.031)
IO2	2	10.597	0.205	(8.521; 12.673)
IO3	2	13.88	1.41	(11.81; 15.96)
IO4	2	22.34	2.12	(20.27; 24.42)
PO1	2	29.03	2.21	(26.95; 31.10)
PC1	2	25.718	1.009	(23.642; 27.793)

Pooled StDev = 1.33373

Tukey Pairwise Comparisons

Grouping Information Using the Tukey Method and 95% Confidence

Factor	N	Mean	Grouping
PO1	2	29.03	A
PC1	2	25.718	A B
IC2	2	24.967	A B

IC3	2	23.648	B C
PT1	2	22.419	B C
IO4	2	22.34	B C
IO1	2	18.955	C D
PT2	2	15.826	D E
IO3	2	13.88	D E
IC1	2	12.654	E
IO2	2	10.597	E

Means that do not share a letter are significantly different.

SUCROSE

Factor Information

Factor	Levels	Values
Factor	11	IC1; IC2; IC3; PT1; PT2; IO1; IO2; IO3; IO4; PO1; PC1

Analysis of Variance

Source	DF	Adj SS	Adj MS	F-Value	P-Value
Factor	10	10.6937	1.06937	12.65	0.000
Error	11	0.9300	0.08455		
Total	21	11.6237			

Model Summary

S	R-sq	R-sq(adj)	R-sq(pred)
0.290768	92.00%	84.73%	68.00%

Means

Factor	N	Mean	StDev	95% CI
IC1	2	0.703	0.480	(0.251; 1.156)
IC2	2	1.7872	0.0347	(1.3347; 2.2397)
IC3	2	1.7668	0.1099	(1.3142; 2.2193)
PT1	2	1.9590	0.1041	(1.5065; 2.4115)
PT2	2	1.6236	0.0694	(1.1711; 2.0761)
IO1	2	1.0796	0.0463	(0.6271; 1.5322)
IO2	2	0.7913	0.0781	(0.3388; 1.2438)
IO3	2	1.249	0.176	(0.797; 1.702)
IO4	2	1.311	0.292	(0.858; 1.763)
PO1	2	3.231	0.729	(2.778; 3.684)
PC1	2	2.3516	0.1215	(1.8991; 2.8042)

Pooled StDev = 0.290768

Tukey Pairwise Comparisons

Grouping Information Using the Tukey Method and 95% Confidence

Factor	N	Mean	Grouping
PO1	2	3.231	A
PC1	2	2.3516	A B
PT1	2	1.9590	B C
IC2	2	1.7872	B C D
IC3	2	1.7668	B C D
PT2	2	1.6236	B C D

IO4	2	1.311	B C D
IO3	2	1.249	B C D
IO1	2	1.0796	C D
IO2	2	0.7913	D
IC1	2	0.703	D

Means that do not share a letter are significantly different.

FRUCTOSE

Factor Information

Factor	Levels	Values
Factor	11	IC1; IC2; IC3; PT1; PT2; IO1; IO2; IO3; IO4; PO1; PC1

Analysis of Variance

Source	DF	Adj SS	Adj MS	F-Value	P-Value
Factor	10	475.69	47.569	43.74	0.000
Error	11	11.96	1.087		
Total	21	487.65			

Model Summary

S	R-sq	R-sq(adj)	R-sq(pred)
1.04283	97.55%	95.32%	90.19%

Means

Factor	N	Mean	StDev	95% CI
IC1	2	11.032	1.145	(9.409; 12.655)
IC2	2	21.974	1.135	(20.351; 23.597)
IC3	2	21.490	0.937	(19.867; 23.113)
PT1	2	18.705	1.085	(17.082; 20.328)
PT2	2	12.597	0.665	(10.974; 14.220)
IO1	2	18.426	1.019	(16.803; 20.049)
IO2	2	9.007	0.218	(7.384; 10.630)
IO3	2	11.680	0.959	(10.057; 13.303)
IO4	2	21.24	1.82	(19.62; 22.87)
PO1	2	21.015	0.606	(19.392; 22.638)
PC1	2	19.581	1.088	(17.958; 21.204)

Pooled StDev = 1.04283

Tukey Pairwise Comparisons

Grouping Information Using the Tukey Method and 95% Confidence

Factor	N	Mean	Grouping
IC2	2	21.974	A
IC3	2	21.490	A
IO4	2	21.24	A
PO1	2	21.015	A
PC1	2	19.581	A
PT1	2	18.705	A
IO1	2	18.426	A
PT2	2	12.597	B
IO3	2	11.680	B

IC1	2	11.032	B
IO2	2	9.007	B

Means that do not share a letter are significantly different.

WATER ACTIVITY

Factor Information

Factor	Levels	Values
Factor	10	IC1; IC2; PT1; PT2; IO1; IO2; IO3; IO4; PO1; PC1

Analysis of Variance

Source	DF	Adj SS	Adj MS	F-Value	P-Value
Factor	9	0.009667	0.001074	1.31	0.293
Error	19	0.015531	0.000817		
Total	28	0.025198			

Model Summary

S	R-sq	R-sq(adj)	R-sq(pred)
0.0285909	38.36%	9.17%	0.00%

Means

Factor	N	Mean	StDev	95% CI
IC1	3	0.7187	0.0297	(0.6841; 0.7532)
IC2	3	0.7310	0.0294	(0.6965; 0.7655)
PT1	3	0.6993	0.0274	(0.6648; 0.7339)
PT2	3	0.6883	0.0310	(0.6538; 0.7229)
IO1	3	0.7030	0.0304	(0.6685; 0.7375)
IO2	3	0.7383	0.0248	(0.7038; 0.7729)
IO3	3	0.7340	0.0252	(0.6995; 0.7685)
IO4	2	0.7450	0.0382	(0.7027; 0.7873)
PO1	3	0.7073	0.0301	(0.6728; 0.7419)
PC1	3	0.6980	0.0221	(0.6635; 0.7325)

Pooled StDev = 0.0285909

Tukey Pairwise Comparisons

Grouping Information Using the Tukey Method and 95% Confidence

Factor	N	Mean	Grouping
IO4	2	0.7450	A
IO2	3	0.7383	A
IO3	3	0.7340	A
IC2	3	0.7310	A
IC1	3	0.7187	A
PO1	3	0.7073	A
IO1	3	0.7030	A
PT1	3	0.6993	A
PC1	3	0.6980	A
PT2	3	0.6883	A

Means that do not share a letter are significantly different.

COLOR: L

Factor Information

Factor	Levels	Values
Factor	11	IC1; IC2; IC3; PT1; PT2; IO1; IO2; IO3; IO4; PO1; PC1

Analysis of Variance

Source	DF	Adj SS	Adj MS	F-Value	P-Value
Factor	10	791.96	79.1964	263.76	0.000
Error	44	13.21	0.3003		
Total	54	805.17			

Model Summary

S	R-sq	R-sq(adj)	R-sq(pred)
0.547954	98.36%	97.99%	97.44%

Means

Factor	N	Mean	StDev	95% CI
IC1	5	65.052	0.526	(64.558; 65.546)
IC2	5	64.1380	0.1867	(63.6441; 64.6319)
IC3	5	67.4780	0.1502	(66.9841; 67.9719)
PT1	5	65.5120	0.0672	(65.0181; 66.0059)
PT2	5	58.060	0.301	(57.566; 58.554)
IO1	5	69.018	0.537	(68.524; 69.512)
IO2	5	66.976	0.421	(66.482; 67.470)
IO3	5	68.612	0.227	(68.118; 69.106)
IO4	5	65.4280	0.1695	(64.9341; 65.9219)
PO1	5	58.180	1.408	(57.686; 58.674)
PC1	5	59.758	0.587	(59.264; 60.252)

Pooled StDev = 0.547954

Tukey Pairwise Comparisons

Grouping Information Using the Tukey Method and 95% Confidence

Factor	N	Mean	Grouping
IO1	5	69.018	A
IO3	5	68.612	A B
IC3	5	67.4780	B C
IO2	5	66.976	C
PT1	5	65.5120	D
IO4	5	65.4280	D
IC1	5	65.052	D E
IC2	5	64.1380	E
PC1	5	59.758	F
PO1	5	58.180	G
PT2	5	58.060	G

Means that do not share a letter are significantly different.

COLOR: a

Factor Information

Factor	Levels	Values
Factor	11	IC1; IC2; IC3; PT1; PT2; IO1; IO2; IO3; IO4; PO1; PC1

Analysis of Variance

Source	DF	Adj SS	Adj MS	F-Value	P-Value
Factor	10	2.76185	0.276185	191.43	0.000
Error	44	0.06348	0.001443		
Total	54	2.82533			

Model Summary

S	R-sq	R-sq(adj)	R-sq(pred)
0.0379832	97.75%	97.24%	96.49%

Means

Factor	N	Mean	StDev	95% CI
IC1	5	2.9280	0.0421	(2.8938; 2.9622)
IC2	5	2.4020	0.0311	(2.3678; 2.4362)
IC3	5	2.29800	0.00837	(2.26377; 2.33223)
PT1	5	2.5020	0.0228	(2.4678; 2.5362)
PT2	5	2.1960	0.0305	(2.1618; 2.2302)
IO1	5	2.6320	0.0383	(2.5978; 2.6662)
IO2	5	2.4480	0.0228	(2.4138; 2.4822)
IO3	5	2.6060	0.0643	(2.5718; 2.6402)
IO4	5	2.37200	0.02168	(2.33777; 2.40623)
PO1	5	2.9080	0.0517	(2.8738; 2.9422)
PC1	5	2.6800	0.0485	(2.6458; 2.7142)

Pooled StDev = 0.0379832

Tukey Pairwise Comparisons

Grouping Information Using the Tukey Method and 95% Confidence

Factor	N	Mean	Grouping
IC1	5	2.9280	A
PO1	5	2.9080	A
PC1	5	2.6800	B
IO1	5	2.6320	B
IO3	5	2.6060	B
PT1	5	2.5020	C
IO2	5	2.4480	C D
IC2	5	2.4020	D
IO4	5	2.37200	D E
IC3	5	2.29800	E
PT2	5	2.1960	F

Means that do not share a letter are significantly different.

COLOR: b

Factor Information

Factor	Levels	Values
Factor	11	IC1; IC2; IC3; PT1; PT2; IO1; IO2; IO3; IO4; PO1; PC1

Analysis of Variance

Source	DF	Adj SS	Adj MS	F-Value	P-Value

Factor	10	461.805	46.1805	1947.05	0.000
Error	44	1.044	0.0237		
Total	54	462.849			

Model Summary

S	R-sq	R-sq(adj)	R-sq(pred)
0.154007	99.77%	99.72%	99.65%

Means

Factor	N	Mean	StDev	95% CI
IC1	5	8.9120	0.1417	(8.7732; 9.0508)
IC2	5	9.0740	0.0896	(8.9352; 9.2128)
IC3	5	6.8240	0.0439	(6.6852; 6.9628)
PT1	5	10.9220	0.0303	(10.7832; 11.0608)
PT2	5	10.2500	0.1162	(10.1112; 10.3888)
IO1	5	5.1720	0.0926	(5.0332; 5.3108)
IO2	5	5.1520	0.1047	(5.0132; 5.2908)
IO3	5	4.7020	0.0712	(4.5632; 4.8408)
IO4	5	5.9080	0.1139	(5.7692; 6.0468)
PO1	5	12.990	0.355	(12.851; 13.129)
PC1	5	12.578	0.229	(12.439; 12.717)

Pooled StDev = 0.154007

Tukey Pairwise Comparisons

Grouping Information Using the Tukey Method and 95% Confidence

Factor	N	Mean	Grouping
PO1	5	12.990	A
PC1	5	12.578	B
PT1	5	10.9220	C
PT2	5	10.2500	D
IC2	5	9.0740	E
IC1	5	8.9120	E
IC3	5	6.8240	F
IO4	5	5.9080	G
IO1	5	5.1720	H
IO2	5	5.1520	H
IO3	5	4.7020	I

Means that do not share a letter are significantly different.

TPC

Factor Information

Factor	Levels	Values
Factor	11	IC1; IC2; IC3; PT1; PT2; IO1; IO2; IO3; IO4; PO1; PC1

Analysis of Variance

Source	DF	Adj SS	Adj MS	F-Value	P-Value
Factor	10	248.099	24.8099	235.27	0.000
Error	22	2.320	0.1055		
Total	32	250.419			

Model Summary

S	R-sq	R-sq(adj)	R-sq(pred)
0.324738	99.07%	98.65%	97.92%

Means

Factor	N	Mean	StDev	95% CI
IC1	3	4.367	0.208	(3.978; 4.755)
IC2	3	3.8333	0.1155	(3.4445; 4.2222)
IC3	3	5.167	0.252	(4.778; 5.555)
PT1	3	9.033	0.586	(8.645; 9.422)
PT2	3	11.033	0.321	(10.645; 11.422)
IO1	3	4.233	0.208	(3.845; 4.622)
IO2	3	4.133	0.208	(3.745; 4.522)
IO3	3	3.1667	0.1528	(2.7778; 3.5555)
IO4	3	3.700	0.265	(3.311; 4.089)
PO1	3	9.100	0.436	(8.711; 9.489)
PC1	3	9.267	0.473	(8.878; 9.655)

Pooled StDev = 0.324738

Tukey Pairwise Comparisons

Grouping Information Using the Tukey Method and 95% Confidence

Factor	N	Mean	Grouping
PT2	3	11.033	A
PC1	3	9.267	B
PO1	3	9.100	B
PT1	3	9.033	B
IC3	3	5.167	C
IC1	3	4.367	C D
IO1	3	4.233	C D
IO2	3	4.133	D
IC2	3	3.8333	D E
IO4	3	3.700	D E
IO3	3	3.1667	E

Means that do not share a letter are significantly different.

ABTS

Factor Information

Factor	Levels	Values
Factor	10	IC1; IC2; IC3; PT2; IO1; IO2; IO3; IO4; PO1; PC1

Analysis of Variance

Source	DF	Adj SS	Adj MS	F-Value	P-Value
Factor	9	8170313284	907812587	455.75	0.000
Error	20	39837771	1991889		
Total	29	8210151054			

Model Summary

S R-sq R-sq(adj) R-sq(pred)

1411.34	99.51%	99.30%	98.91%

Means

Factor	N	Mean	StDev	95% CI
IC1	3	2.7589	0.955	(2.5889; 2.9288)
IC2	3	2.6009	0.268	(2.4309; 2.7709)
IC3	3	6.9568	0.1054	(6.7868; 7.1268)
PT2	3	6.5980	0.1642	(6.4280; 6.7680)
IO1	3	3.6438	0.1181	(3.4738; 3.8138)
IO2	3	4.4215	0.1203	(4.2515; 4.5914)
IO3	3	3.9790	0.2771	(3.8090; 4.1490)
IO4	3	3.6323	0.1549	(3.4624; 3.8023)
PO1	3	1.4108	0.484	(1.2408; 1.5808)
PC1	3	2.8947	0.1406	(2.7247; 3.0647)

Pooled StDev = 1411.34

Tukey Pairwise Comparisons
Grouping Information Using the Tukey Method and 95% Confidence

Factor	N	Mean	Grouping
IC3	3	6.9568	A
PT2	3	6.5980	A
IO2	3	4.4215	B
IO3	3	3.9790	C
IO1	3	3.6438	C
IO4	3	3.6323	C
PC1	3	2.8947	D
IC1	3	2.7589	D
IC2	3	2.6009	D
PO1	3	1.4108	E

Means that do not share a letter are significantly different.

DPPH

Factor Information

Factor	Levels	Values
Factor	11	IC1; IC2; IC3; PT1; PT2; IO1; IO2; IO3; IO4; PO1; PC1

Analysis of Variance

Source	DF	Adj SS	Adj MS	F-Value	P-Value
Factor	10	259171892	25917189	2.63	0.030
Error	21	206735345	9844540		
Total	31	465907236			

Model Summary

S	R-sq	R-sq(adj)	R-sq(pred)
3137.60	55.63%	34.50%	0.00%

Means

Factor N Mean StDev 95% CI

IC1	3	0.6547	0.2203	(0.2780; 1.0314)
IC2	3	0.5065	0.2967	(0.1298; 0.8832)
IC3	3	0.6597	0.2379	(0.2830; 1.0365)
PT1	3	1.1437	0.7955	(0.7669; 1.5204)
PT2	3	1.3277	0.2679	(1.510; 1.1044)
IO1	3	0.4611	0.2461	(0.2844; 0.8378)
IO2	3	0.5259	0.1871	(0.1491; 0.9026)
IO3	3	0.8594	0.1084	(0.4826; 1.2361)
IO4	3	0.5670	0.1096	(0.1903; 0.9437)
PO1	2	0.3179	0.1812	(0.1435; 0.7793)
PC1	3	0.63101	0.1128	(0.25429; 1.00773)

Pooled StDev = 3.13760

Tukey Pairwise Comparisons

Grouping Information Using the Tukey Method and 95% Confidence

Factor	N	Mean	Grouping
PT2	3	1.3277	A
PT1	3	1.1437	A
IO3	3	0.8594	A
IC3	3	0.6597	A
IC1	3	0.6547	A
PC1	3	0.6310.1	A
IO4	3	0.5670	A
IO2	3	0.5259	A
IC2	3	0.5065	A
IO1	3	0.4611	A
PO1	2	0.3179	A

Means that do not share a letter are significantly different.

ORAC

Factor Information

Factor	Levels	Values
Factor	11	IC1; IC2; IC3; PT1; PT2; IO1; IO2; IO3; IO4; PO1; PC1

Analysis of Variance

Source	DF	Adj SS	Adj MS	F-Value	P-Value
Factor	10	0.163292	0.016329	91.01	0.000
Error	22	0.003947	0.000179		
Total	32	0.167239			

Model Summary

S	R-sq	R-sq(adj)	R-sq(pred)
0.0133950	97.64%	96.57%	94.69%

Mean

Factor	N	Mean	StDev	95% CI
IC1	3	10.956	0.420	(9.352; 12.560)
IC2	3	10.331	0.514	(8.727; 11.935)
IC3	3	13.359	0.916	(11.756; 14.963)

PT1	3	25.11	1.79	(23.51; 26.72)
PT2	3	21.6732	0.1262	(20.0694; 23.2771)
IO1	3	6.63	2.29	(5.02; 8.23)
IO2	3	10.701	0.271	(9.097; 12.305)
IO3	3	8.063	1.611	(6.459; 9.667)
IO4	3	8.87	2.20	(7.27; 10.47)
PO1	3	25.927	0.323	(24.323; 27.531)
PC1	3	22.935	1.549	(21.331; 24.539)

Pooled StDev = 0.0133950

Tukey Pairwise Comparisons

Grouping Information Using the Tukey Method and 95% Confidence

Factor	N	Mean	Grouping
PO1	3	25.927	A
PT1	3	25.11	A B
PC1	3	22.935	A B
PT2	3	21.6732	B
IC3	3	13.359	C
IC1	3	10.956	C D
IO2	3	10.701	C D
IC2	3	10.331	C D E
IO4	3	8.87	D E
IO3	3	8.063	D E
IO1	3	6.63	E

Means that do not share a letter are significantly different.

Principal Component Analysis: pH; Ash; Moisture; Proteins; Glucose; Sucrose; Fructuose; Water

Eigenanalysis of the Correlation Matrix

18 cases used, 37 cases contain missing values

Eigenvalue	6.1674	3.8969	1.7764	1.3254	0.8891	0.7551	0.5305	0.2222	0.1808	0.1224
Proportion	0.385	0.244	0.111	0.083	0.056	0.047	0.033	0.014	0.011	0.008
Cumulative	0.385	0.629	0.740	0.823	0.878	0.926	0.959	0.973	0.984	0.992

Eigenvalue	0.0782	0.0402	0.0126	0.0020	0.0008	0.0001
Proportion	0.005	0.003	0.001	0.000	0.000	0.000
Cumulative	0.997	0.999	1.000	1.000	1.000	1.000

Variable	PC1	PC2	PC3	PC4	PC5	PC6	PC7	PC8	PC9
pH	0.242	0.350	0.039	0.113	0.048	0.190	-0.174	-0.402	-0.390
Ash	-0.020	-0.225	-0.240	0.477	-0.501	-0.064	0.536	-0.204	-0.150
Moisture	-0.124	0.201	0.283	-0.559	-0.221	-0.256	0.282	0.208	-0.425
Proteins	0.330	0.040	-0.266	0.088	-0.275	-0.168	-0.277	0.395	0.114
Glucose	0.281	-0.309	-0.121	-0.182	0.112	0.250	0.083	-0.021	-0.168

Sucrose	0.339	-0.164	0.099	-0.201	0.054	0.183	0.018	-0.147	0.455
Fructuose	0.192	-0.336	-0.274	-0.179	0.221	0.313	0.051	0.028	-0.428
Water Activity	-0.169	-0.139	-0.049	-0.358	-0.676	0.288	-0.393	-0.279	0.036
L	-0.380	-0.121	0.007	0.061	0.120	0.106	-0.030	-0.179	-0.083
a	0.028	-0.259	0.483	0.327	-0.004	-0.232	-0.454	-0.158	-0.254
b	0.376	-0.046	0.167	0.052	-0.168	-0.026	0.042	0.242	-0.188
TPC	0.352	0.196	0.101	-0.022	0.040	-0.115	0.108	-0.344	-0.085
Flavonoids	0.031	-0.130	-0.537	-0.192	0.110	-0.636	-0.251	-0.213	-0.114
ABTS+	-0.063	0.462	-0.231	-0.066	0.045	0.039	0.089	-0.310	0.096
DPPH	0.066	0.417	-0.221	0.206	-0.117	0.271	-0.228	0.288	-0.173
ORAC	0.371	0.057	0.163	-0.087	-0.171	-0.175	0.134	-0.197	0.215

Variable	PC10	PC11	PC12	PC13	PC14	PC15	PC16
pH	0.217	-0.580	0.159	-0.053	-0.018	-0.101	0.009
Ash	0.198	0.020	0.058	0.074	0.042	0.081	-0.015
Moisture	0.351	0.035	-0.047	0.042	0.015	0.076	0.007
Proteins	0.214	-0.260	-0.500	-0.155	-0.130	0.221	0.087
Glucose	0.080	0.141	-0.042	-0.064	0.127	-0.159	0.770
Sucrose	0.574	0.103	0.271	0.138	-0.157	0.234	-0.173
Fructuose	-0.083	0.047	-0.308	0.197	0.012	0.051	-0.514
Water Activity	-0.204	0.027	0.039	-0.035	0.021	0.041	-0.018
L	0.284	0.148	-0.238	-0.454	-0.610	-0.183	-0.011
a	0.156	0.253	-0.183	0.322	0.071	0.091	0.065
b	-0.362	0.049	0.336	0.113	-0.663	-0.040	0.035
TPC	-0.237	0.345	-0.097	-0.518	0.096	0.457	-0.056
Flavonoids	0.065	0.089	0.304	0.006	-0.057	-0.105	-0.025
ABTS+	-0.072	0.185	-0.326	0.555	-0.293	0.139	0.220
DPPH	0.240	0.550	0.206	-0.055	0.139	-0.206	-0.102
ORAC	-0.029	0.091	-0.305	-0.013	0.066	-0.721	-0.190
